# Unraveling the Interfacial Properties of Twisted Single‐Crystal Au(111)/MoS_2_ Heterostructures: A Pathway to Robust Superlubricity

**DOI:** 10.1002/advs.202415884

**Published:** 2025-04-04

**Authors:** Yuanpeng Yao, Yiming Song, Bozhao Wu, Sebastian Scherb, Shuyu Huang, Antoine Hinaut, Thilo Glatzel, Ernst Meyer, Ze Liu, Wengen Ouyang

**Affiliations:** ^1^ Department of Engineering Mechanics School of Civil Engineering Wuhan University Wuhan Hubei 430072 P. R. China; ^2^ Department of Physics University of Basel Klingelbergstrasse 82 Basel 4056 Switzerland; ^3^ College of Science Wuhan University of Science and Technology Wuhan 430081 P. R. China; ^4^ Hubei Province Key Laboratory of Systems Science in Metallurgical Process College of Science Wuhan University of Science and Technology Wuhan 430081 P. R. China; ^5^ State Key Laboratory of Water Resources Engineering and Management Wuhan University Wuhan Hubei 430072 P. R. China

**Keywords:** Au(111)/MoS_2_, kelvin probe force microscopy, moiré superlattice, semi‐anisotropic interfacial potential, superlubricity

## Abstract

A comprehensive study of monolayer MoS_2_ on a single‐crystal Au(111) surface is reported, combining ultra‐high vacuum scanning probe microscopy with advanced computational methods. Kelvin probe force microscopy precisely quantified the work function of the heterointerface, while topographic analysis by contact and non‐contact atomic force microscopy revealed a moiré superlattice with an interfacial twist angle of 0.45° between MoS_2_ and Au(111). To accurately model and predict the twist angle and out‐of‐plane corrugation of these moiré superlattices, a semi‐anisotropic interlayer force field based on density functional theory is developed. This avoids the limitations of conventional pairwise potentials, and our results show excellent agreement with experiments. Furthermore, friction simulations revealed a non‐monotonic dependence on the interfacial twist angle, with small angles exhibiting unexpectedly large shear stress, suggesting that MoS_2_ could serve as an effective superlubric coating for gold. This work establishes a robust framework for the investigation of van der Waals heterostructures, bridging nanoscale experimental observations with first‐principles calculations, and providing insights for the design of novel nanoscale devices with tailored electronic, mechanical, and tribological properties.

## Graphical abstract

Interfacial mechanical properties of MoS_2_/Au(111) heterostructures are revealed by advanced experimental and computational methods. The developed semi‐anisotropic interlayer potential (SAIP) accurately predicts moiré‐driven out‐of‐plane corrugation, aligning with experimental observations. Friction simulations demonstrate a non‐monotonic shear stress dependence, with unexpectedly high friction at small twist angles (<5°), which positions MoS_2_ as a versatile material for ultra‐low‐wear coatings in nanomechanical systems.

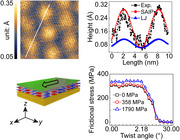

## 1 Introduction

Transition metal dichalcogenides (TMDCs) consist of a layer of transition metal atoms sandwiched between two layers of chalcogen atoms. These layered structures are held together by weak van der Waals (vdW) forces that facilitate lamellar shear between adjacent TMDC layers.^[^ ^1^ ^]^ Due to their exceptional mechanical, electronic, and frictional properties, TMDCs have attracted significant attention in both advanced technological applications and fundamental scientific research.^[^ ^2^, ^3^, ^4^, ^5^, ^6^, ^7^, ^8^, ^9^, ^10^ ^]^ Monolayer MoS_2_, a remarkably stable two‐dimensional (2D) semiconductor within the TMDC family, has emerged as a research hotspot due to its broad prospects in areas such as field‐effect transistors, photodetectors, integrated circuits, and sensors, among others.^[^ ^11^, ^12^, ^13^, ^14^ ^]^ So far, the preparation of monolayer MoS_2_ primarily involves two methods: mechanical exfoliation^[^ ^12^, ^15^, ^16^, ^17^, ^18^, ^19^ ^]^ and chemical vapor deposition (CVD).^[^ ^20^, ^21^, ^22^, ^23^, ^24^ ^]^ While mechanical exfoliation is popular for producing high‐quality monolayers for fundamental research, CVD has gained particular attention due to its excellent controllability and scalability,^[^ ^25^, ^26^, ^27^, ^28^ ^]^ especially when using Au(111) as the substrate.^[^ ^29^, ^30^, ^31^, ^32^ ^]^ A critical consideration in experiments is the purity of the prepared MoS_2_. High purity MoS_2_ layers are essential, as defects can significantly affect the measurement results of the intrinsic properties of 2D materials, such as mechanical,^[^ ^33^ ^]^ thermal^[^ ^33^ ^]^ and electronic properties.^[^ ^33^ ^]^

The Au/MoS_2_ interface presents a compelling frontier in nanomaterial research, offering insights into both synthesis optimization and interfacial dynamics. Direct observation of this interface is paramount for elucidating the nature of Au‐MoS_2_ interactions, particularly given the mobile character of Au and the potential for dynamic interfacial evolution.^[^ ^34^ ^]^ Recent studies have reported the behavior of nanostructured gold on 2D materials,^[^ ^35^, ^36^, ^37^, ^38^ ^]^ revealing the effect of chemical synthesis and aggregation of Au nanoparticles on CVD graphene or mechanically exfoliated MoS_2_.^[^ ^35^, ^36^, ^37^ ^]^ However, fundamental questions persist regarding the governing principles of interface evolution, including the relationship between Au and MoS_2_ lattices and the atomic‐scale transfer pathways of Au. Understanding these substrate‐metal interactions and their impact on the intrinsic properties of MoS_2_ is crucial for advancing both fundamental science and potential applications.^[^ ^39^, ^40^, ^41^, ^42^, ^43^ ^]^ While experimental observation of interface evolution is challenged by external perturbations, molecular dynamics (MD) simulations offer a powerful alternative for probing these nanoscale phenomena. Yet, the current lack of accurate force fields for the Au‐MoS_2_ interlayer interactions has limited the scope of MD studies in this system. Addressing this gap could unlock new insights into the Au(111)/MoS_2_ interface, potentially revolutionizing our approach to designing and optimizing these hybrid nanomaterials for next‐generation devices and applications.

In this work, we demonstrate the successful growth of single‐crystal monolayer MoS_2_ on Au(111) substrates under ultra‐high vacuum (UHV) conditions, followed by comprehensive characterization using advanced scanning probe microscopy techniques. Non‐contact atomic force microscopy (nc‐AFM) reveals an intriguing out‐of‐plane corrugation of MoS_2_, induced by a moiré superlattice at a twist angle of 0.45° between the contacting surfaces. To accurately mimic the vdW interaction between Au and MoS_2_, we develop a semi‐anisotropic interfacial potential (SAIP) based on state‐of‐art density functional theory (DFT) calculations. This computational approach shows remarkable consistency with experimental results across multiple measurements. DFT calculations of the MoS_2_ work function on gold exhibit excellent agreement with Kelvin probe force microscopy (KPFM) measurements. Moreover, the SAIP predicts an out‐of‐plane moiré corrugation that closely matches nc‐AFM observations, underscoring the accuracy and versatility of our computational framework.

Notably, our simulations of the frictional properties of the Au/MoS_2_ heterostructure reveal an unexpected non‐monotonic dependence on the interfacial twist angle. This finding opens new avenues for tuning the tribological properties of 2D material‐metal interfaces through precise control of their relative orientation. The high interfacial shear stress observed in the Au(111)/MoS_2_ heterojunction indicates that MoS_2_ is a promising candidate for robust superlubric coating on gold surfaces, leveraging its ability to exhibit superlubric behavior with various materials.^[^ ^44^, ^45^, ^46^ ^]^ Macroscale tribological experiments^[^ ^47^, ^48^ ^]^ have revealed that the incorporation of MoS_2_ into Au reduces friction and extends lifespan with minimal impact on electrical conductivity.^[^ ^49^ ^]^ These results not only advance our fundamental understanding of 2D material‐metal interfaces but also pave the way for designing novel nanoscale devices with tailored electronic and tribological properties. Our work provides crucial insights into the growth, characterization, and modeling of 2D material heterostructures, offering a comprehensive approach.

## 2 Results and Discussion

### 2.1 Experimental Characterization

**Figure 1:**
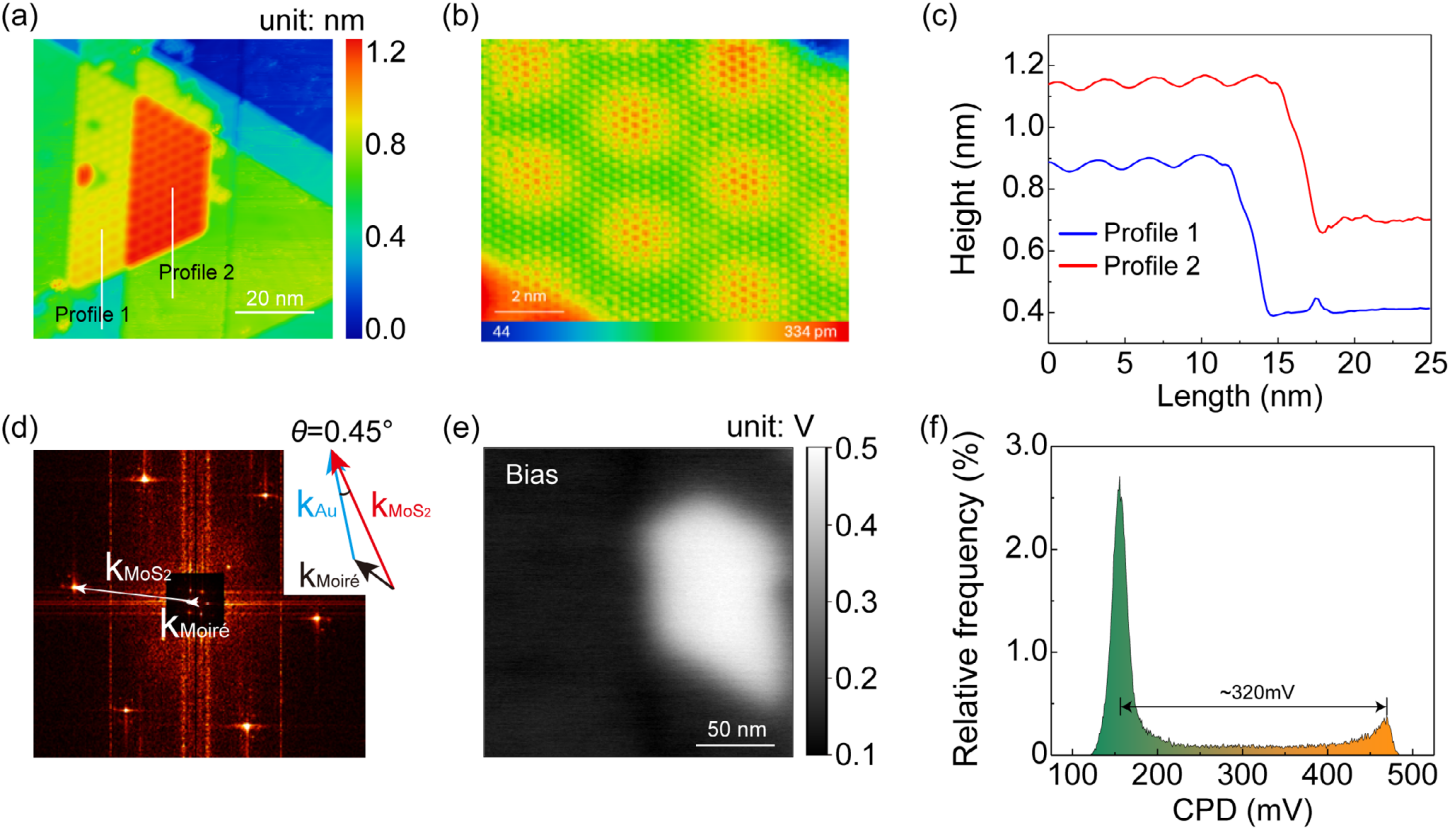
Characterization of monolayer MoS_2_ grown on an Au(111) substrate under UHV conditions. a) STM image of a monolayer MoS_2_ flake with dimensions of 78 nm × 78 nm. b) Atom‐resolved STM image of the atomic lattice and moiré superstructure via CO functionalized tip. c) Line profiles across the MoS_2_ island shown in (a). d) 2D fast Fourier transform (FFT) of STM image of the MoS_2_ sheet on Au (111). e) KPFM image. The bright and dark regions represent MoS_2_ and bare Au(111), respectively. f) Histogram of the KPFM image, showing the difference in surface potential of the respective MoS_2_ flake and Au(111) surface is ∼320 mV.

**Figure** **1**a illustrates the topography of a typical epitaxial grown MoS_2_ flake across an Au(111) step edge, as imaged by scanning tunnel microscope (STM) at 5 K. The contour along the white line drawn across the two gold terraces indicates that the MoS_2_ has monolayer height of ∼0.51 nm (Figure 1c). Atomically resolved STM imaging with a CO‐functionalized tip (Figure 1b) reveals the MoS_2_ atomic lattice, exhibiting a periodicity of ∼3.15 Å, superimposed with a long‐range periodic moiré pattern with a periodicity of ∼3.3 nm.^[^ ^50^, ^51^ ^]^ In addition, high‐resolution structural characterization of MoS_2_ surface was performed using nc‐AFM at room temperature (Figure S1a,b and Section S1, Supporting Information). Regarding the nc‐AFM working at room temperature, the tip‐sample distance was controlled based on the frequency shift of the second flexural oscillation of the cantilever, ensuring stable and high‐resolution scanning conditions.^[^ ^50^ ^]^ Figure S1a,b (Section S1 of the Supporting Information) illustrates a flawlessly detailed atomic lattice and superstructure of MoS_2_, providing evidence of the meticulous quality in sample preparation. For comparison, Figure S1c (Supporting Information) shows images of regions of defective MoS_2_. Notably, we observed only one type of superstructure arrangement, consistent with our previous experimental measurements and those reported in literature.^[^ ^52^, ^53^, ^54^, ^55^ ^]^ Given a typical sulfur vacancy density of 2 × 10^11^ cm^−2[^ ^56^ ^]^ and our scanning area of 20 × 20 nm^2^, we expect less than one defect per scan. Thus, the MoS_2_ surface can be considered nearly defect‐free.

### 2.2 Optimal interfacial Angle of the Au(111)/MoS_2_ heterostructure

**Figure 2:**
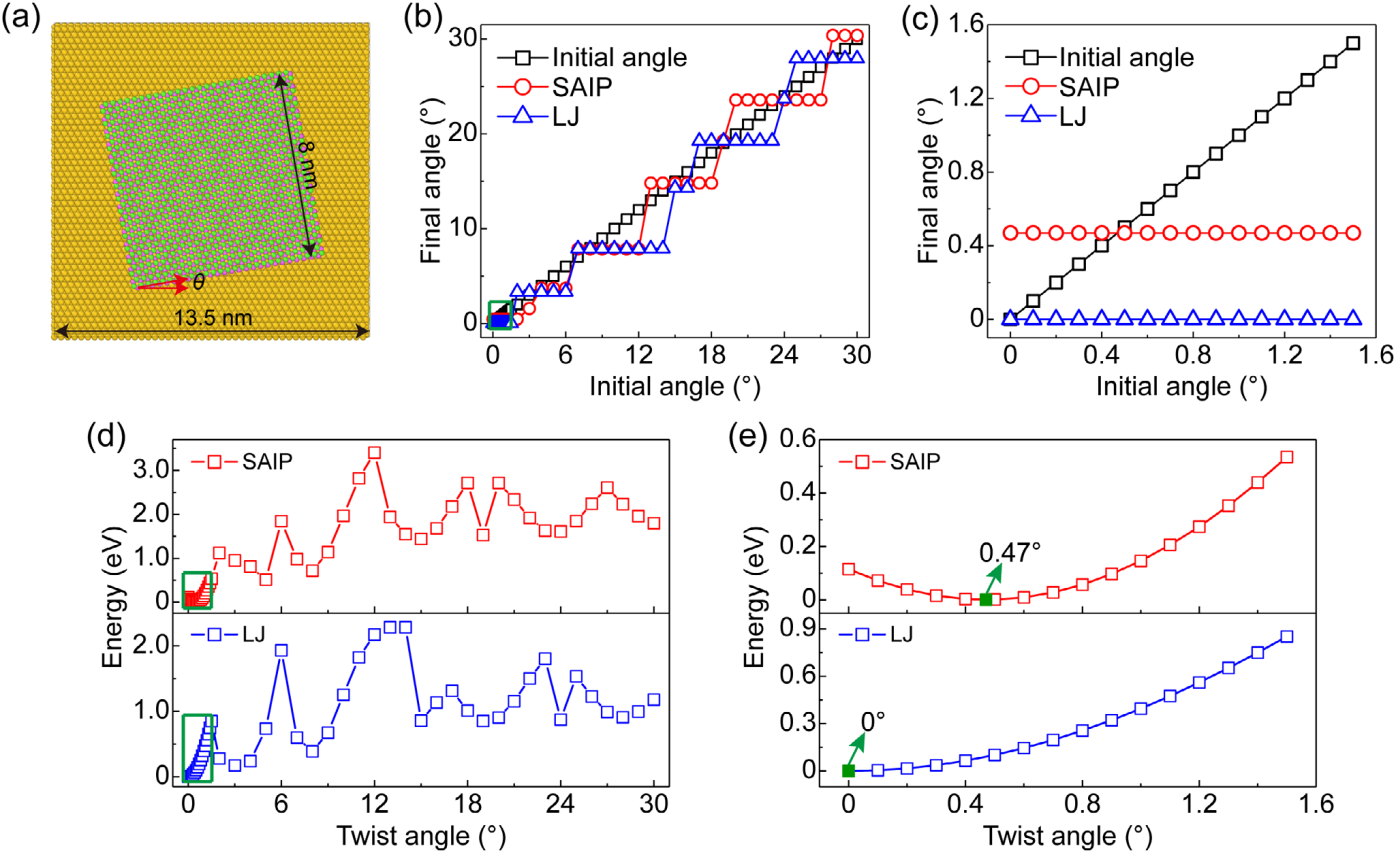
Structural and energetic analysis of the Au(111)/MoS_2_ heterostructure. a) Schematic diagram of Au(111)/MoS_2_ heterostructure. The dimensions of MoS_2_ and the Au substrate are 8 nm × 8 nm and 13.5 nm × 13.5 nm, respectively. ***θ*** represents the twist angle between Au and MoS_2_. b) Twist angles ***θ*** extracted from the fully relaxed Au(111)/MoS_2_ heterostructure using SAIP (red line) and LJ potential (blue line), compared to the initial twist angle (black line). c) Magnified view of the region marked by a green rectangle in (b). d) Total energy as a function of twist angle, with the minimum value set as the reference point. Red and blue lines represent the SAIP (Table S2, Supporting Information) and LJ calculation results,^[^ ^60^, ^61^ ^]^ respectively. e) Magnified view of the region marked by a green rectangle in panel (d), the green arrow indicates the twist angle corresponding to the minimum energy.

The experimental results of this study indicate that MoS_2_ is not perfectly aligned with the substrate lattice, exhibiting a slight twist angle of ∼0.45°, as determined from the FFT image (Figure 1d), a phenomenon previously reported in the literature.^[^ ^44^, ^52^ ^]^ This misalignment indicates that a slight twist might be energetically more favorable.^[^ ^57^ ^]^ To elucidate the underlying mechanism, we investigated the specific impact of the equilibrium misalignment angle on the energy through MD simulations. However, we encountered a challenge in accurately characterizing the vdW interlayer interactions in Au/MoS_2_ heterostructures. The commonly used Lennard‐Jones (LJ) potential fails to accurately capture the anisotropic vdW interlayer interactions,^[^ ^58^ ^]^ resulting in discrepancies between simulation and experimental results (blue lines in **Figure** **2**b,c). To address this issue, we introduced the SAIP^[^ ^58^, ^59^ ^]^ to depict the vdW interactions between Au and MoS_2_ (see details for parameterization and validation of SAIP in Section S2, Supporting Information). Based on the SAIP, the fully relaxed Au(111)/MoS_2_ heterostructure exhibits an interfacial twist angle of 0.47° when the initial twist angle is in the range of 0° and 2° (Figure 2b,c). This result closely matches our experimental observations, highlighting the superiority of SAIP in describing vdW interlayer interactions between Au and MoS_2_.

To further explore the relationship between twist angle and system energy, we expanded our SAIP‐based simulations to cover initial twist angles ranging from 0° to 30°. This broader investigation revealed a complex landscape of relaxed angles corresponding to different initial configurations (Figure 2b,c). We identified several distinct ranges of initial twist angles that resulted in specific relaxed angles: 0°–2° (0.47°), 2.1°–3° (1.58°), 4°–6° (3.74°), 7°–12° (7.85°), 13°–18° (14.81°), 19° (19.29°), 20°–27° (23.58°), and 28°–30° (30.39°). These relaxed angles correspond to local minima in the energy landscape of the system (Figure 2d), with the global minimum energy occurring at a twist angle of 0.47° (Figure 2e). This energy landscape provides a compelling explanation for our experimental observation of a ∼0.45° twist angle in the Au(111)/MoS_2_ heterostructure. The system naturally settles into this configuration as it represents the lowest energy state and, consequently, the most stable arrangement.

To gain deeper insights into the energetics governing this behavior, we analyzed the components of the total system energy (Figure S7, Supporting Information). The total energy comprises two primary contributions: interlayer energy (arising from vdW interactions between Au and MoS_2_) and intralayer energy (from within the MoS_2_ layer and Au substrate). Interestingly, these two components exhibit opposing trends as a function of twist angle, suggesting that the observed equilibrium configuration results from a delicate balance between these competing influences. To investigate the effect of temperature on the stability of the optimal twist angle, we performed additional molecular dynamics simulations, increasing the temperature of the MoS_2_/Au system from 300 to 500 K (see Figure S8 in Section S3, Supporting Information). Our results indicate that the optimal twist angle remains stable at 0.47° within a temperature range of 300 K–450 K. However, beyond this critical temperature (∼450 K), the MoS_2_ layer undergoes noticeable rotation, disrupting the optimal twist angle.

### 2.3 Contact Potential Difference

It is important to note that the accuracy of our MD simulations is fundamentally determined by the DFT dataset used to parameterize the SAIP. In this study, we employed the nonlocal many‐body dispersion (MBD‐NL) method augmented Perdew–Burke–Ernzerhof (PBE) functional to quantify the potential difference between the Au/MoS_2_ and bare Au surfaces, the calculated value (∼315 mV) aligned well with the KPFM measurements. As demonstrated in Figure 1e,f, the MoS_2_ island is reflected in the contact potential difference (CPD) image with clear edges, forming a distinct contrast in surface potential (∼320 mV) compared to the bare Au region (see more details about the KPFM measurements in Experimental Section of main text). The consistency between theoretical calculations and experimental results not only validates the accuracy of our DFT calculations but also lends credence to the reliability of our MD simulations for the Au/MoS_2_ heterostructure. This agreement underscores the robustness of our computational approach in capturing the complex electronic and structural properties of this interface.

### 2.4 Out‐of‐Plane Corrugation

**Figure 3:**
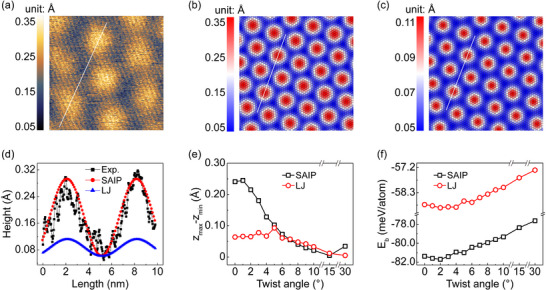
Experimental and simulated moiré superlattice structures of Au(111)/MoS_2_ heterostructure. a) nc‐AFM topographic image of the monolayer MoS_2_ grown on Au(111) substrate, exhibiting the moiré superlattice. Experimental parameters: Δ*f_1st_* = ‐60 Hz, *A_1st_* = 5 nm. b,c) Calculated moiré superlattice of Au(111)/MoS_2_ with a twist angle of 0.45° using SAIP (b) and LJ potential (c). d) Height profiles along the white lines in (a), (b) and (c), corresponding to black (experiment), red (SAIP), and blue (LJ) lines, respectively. e,f) Variation of the out‐of‐plane corrugation of MoS_2_ (e) and BE (f) with twist angle. ***z***_**max**_ and ***z***_**min**_ represent the maximum and minimum values of the *z* coordinates of Mo layer. In panels (e,f), black and red lines represent the calculations using SAIP and LJ potential, respectively.

Utilizing this newly developed SAIP, we thoroughly investigated the out‐of‐plane corrugation of MoS_2_ induced by the natural lattice mismatch between the overlayer and the substrate. Within a moiré unit cell, the atoms of the MoS_2_ overlayer occupy different positions relative to the substrate, giving rise to a long‐range superlattice structure. The out‐of‐plane corrugation of MoS_2_ extracted from the fully relaxed Au(111)/MoS_2_ heterostructure successfully reproduces the experimental nc‐AFM characterization, showing an average height difference of 0.24 ± 0.04 Å (**Figure** **3**a,b,d). Note that the primary scanning parameters affecting the nc‐AFM measurements are amplitude of oscillation *A* and frequency shift Δ*f*. In Figure 3, the cantilever was excited at its first resonance mode, using scanning parameters of Δ*f_1st_* = −60 Hz and *A_1st_* = 5 nm. To investigate the effect of scanning parameters on the measured height profiles, we performed additional nc‐AFM measurements, exciting the cantilever at its second resonance mode (Δ*f_2nd_* = −25 Hz and *A_2nd_* = 400 pm, see Figure S9 in Section S4, Supporting Information). The results demonstrate that the influence of scanning parameters on the nc‐AFM height profiles is negligible. More details and data are presented in Section S4 (Supporting Information).

In contrast, simulation results derived from LJ potentials^[^ ^60^, ^61^ ^]^ show significant discrepancies compared to experimental results (Figure 3c,d; Figure S9c,d, Supporting Information). The consistency between SAIP and experimental results mutually confirms their accuracy. Furthermore, the SAIP is employed to predict the variation of out‐of‐plane corrugation as well as binding energy (BE) across a wide range of twist angles from 0° to 30° (Figure 3e,f). For small twist angles, MoS_2_ exhibits noticeable out‐of‐plane corrugation. As the twisting angle increases, the corrugation gradually decreases, eventually leading to a nearly flat MoS_2_ configuration with minimal out‐of‐plane corrugation (Figure S5, Supporting Information illustrates the moiré superlattices for several typical angles). Notably, at a twist angle of 30°, a special aligned lattice state emerges where a second‐order moiré pattern induces a slightly higher fluctuations in the corrugation (see Section S2.3, Supporting Information for detailed explanation). The calculated BE shows subtle variations across different twist angles, with a maximum difference of ∼4 meV atom^−1^. This gentle variation suggests that while the twist angle does influence the thermodynamic stability of the Au(111)/MoS_2_ heterostructure, its impact is relatively small but potentially significant for fine‐tuning interfacial properties.

### 2.5 Frictional Behavior of Au/MoS_2_ heterointerface

**Figure 4:**
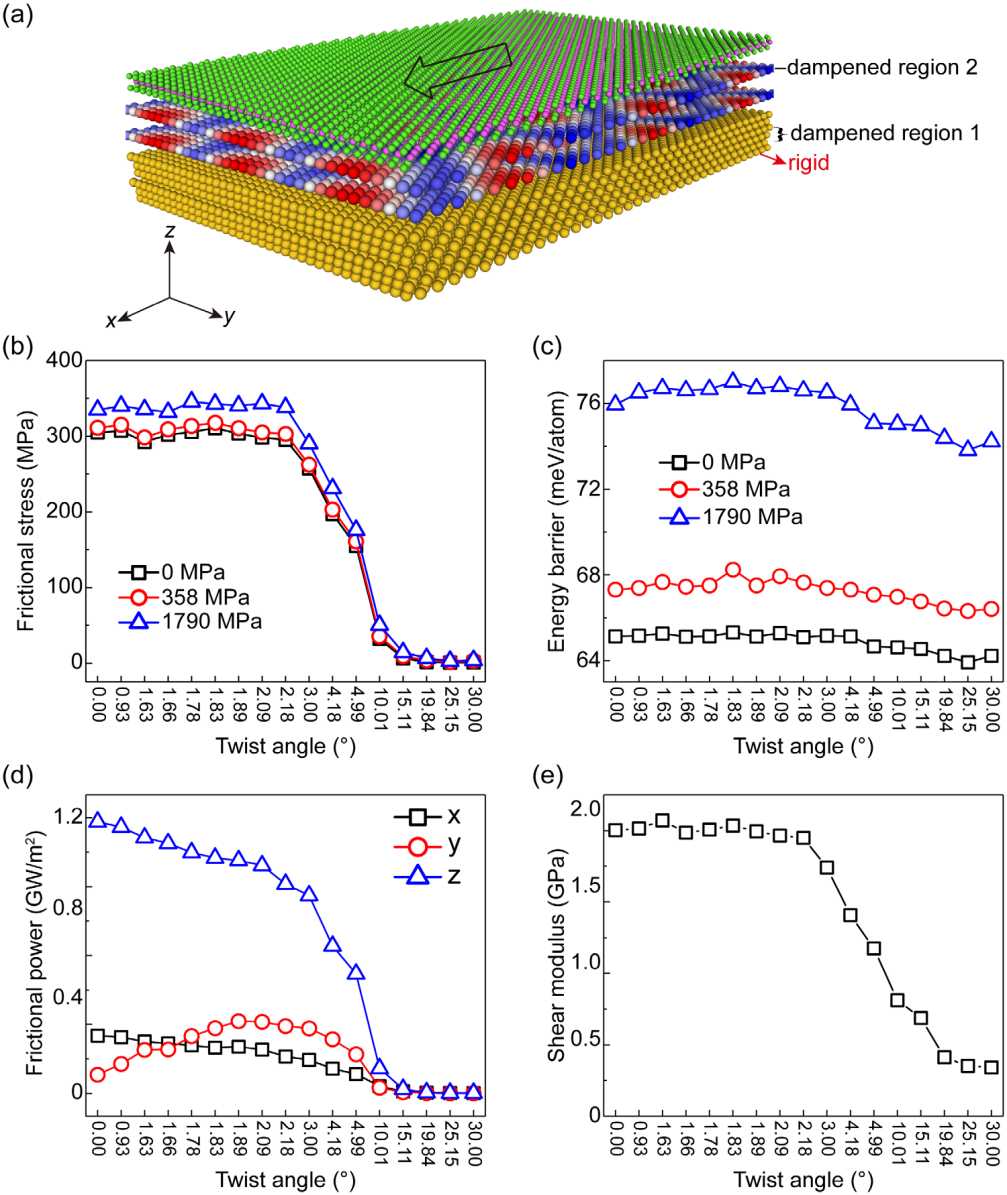
Simulated frictional behavior of Au(111)/MoS_2_ heterojunctions. a) Schematics of the simulation set‐up. Yellow, green, and pink spheres represent Au, S, and Mo atoms, respectively. The red, blue, and white spheres with moiré structure between Au and MoS_2_ represent different out‐of‐plane corrugation of the MoS_2_ layer. b,c) Interfacial frictional stress (b) and sliding barrier (c) between Au(111) and MoS_2_ as a function of twist angle under normal loads of 0 (black), 358 (red), and 1790 MPa (blue), respectively. d) Frictional power of Au(111)/MoS_2_ heterostructure as a function of twist angle along the *x* (black line), *y* (red line), and *z* (blue line) directions. e) Twist‐angle dependence of shear modulus of Au(111)/MoS_2_ heterostructure.

Experimental observations reveal that monolayer MoS_2_ exhibits significant resistance to lateral movement on Au(111) substrate, indicating a strong interfacial friction. We conducted MD simulations to assess the tribological behavior between Au and MoS_2_ using SAIP. The constructed MD model consists of a 1.45 nm‐thick Au substrate and 3‐layer MoS_2_ (**Figure** **4**a; Section S6.1, Supporting Information provided more detailed information). In these simulations, the overall frictional stress, σ*_f_*, that develops during the sliding motion can be written as a sum of the contributions of the different degrees of freedom (DOF) within the Au substrate and MoS_2_ layer to the energy dissipation (the simulation details are provided in Section S6.2, Supporting Information):

(1) $$\sigma_{f}=\frac{\sum_{i=1}^{N_{\mathrm{layer}}}\left[\sum_{k=1}^{N_{i}}m_{k}\sum_{\alpha =x,y,z}\eta_{\alpha }\langle {\left(v_{\alpha ,k}^{i}\left(t\right)-v_{\alpha ,\mathrm{com}}^{i}\left(t\right)\right)}^{2}\rangle +M_{i}\sum_{\alpha =x,y,z}\eta_{\alpha }\langle {\left(v_{\alpha ,\mathrm{com}}^{i}\left(t\right)\right)}^{2}\rangle \right]}{A\cdot v}$$

where *N_i_*, $v_{\mathrm{com}}^{i}$, *M_i_* and *A* are the total number of atoms in the *i*‐th layer, its center‐of‐mass velocity, its total mass and contact area, respectively. *m_k_* represents the atomic masses of S, Mo or Au. *η*_*α*_ is the damping coefficient that characterizes the kinetic energy dissipation rate in the *α* direction (*α* = *x*, *y*, *z*), and 〈 · 〉 denotes a steady‐state time average. According to the MD simulation results, Au(111)/MoS_2_ with aligned lattices exhibits strong interlayer friction stress (∼303 MPa, Figure 4b), significantly higher than widely studied homo‐/hetero‐ structures such as graphite/*h*‐BN,^[^ ^62^ ^]^ graphene/graphene^[^ ^63^ ^]^ and MoS_2_/MoS_2_.^[^ ^10^, ^64^ ^]^ Previous studies have shown that the Au substrate may form a quasi‐covalent bond with the adjacent S atomic layer,^[^ ^65^ ^]^ which we hypothesize is the primary reason for the pronounced interlayer friction in Au(111)/MoS_2_. The strength of interfacial friction is further reflected in the interlayer shear modulus and the sliding energy barrier. The interlayer shear modulus quantifies the resistance to relative sliding between layers, while the potential energy corrugation affects the energy barriers that must be overcome during sliding, both contributing to frictional behavior. Our results indicate that the Au(111)/MoS_2_ heterostructure possesses a significant interlayer shear modulus (∼1.60 GPa) and sliding energy barrier (∼65.13 meV atom^−1^) (Figure 4c,e), consistent with the observed strong frictional effects. Despite the high interfacial friction, the superior tribological performance of the MoS_2_ surface,^[^ ^45^, ^66^, ^67^ ^]^ combined with its strong adhesion to the Au substrate, suggests that MoS_2_ could serve as promising candidates for achieving robust, large‐scale superlubricity coatings.

According to well‐established Novaco‐McTague^[^ ^68^ ^]^ (NM) theory, the equilibrium misalignment angle between the overlayer and substrate lattice is generally nonzero and equal to

(2) $$\theta_{\mathrm{NM}}=\arccos\left[\frac{1+\rho^{2}\left(1+2\delta \right)}{\rho \left(2+\delta \left(1+\rho^{2}\right)\right)}\right]$$

where *ρ* = *a*_s_ /*a*_o_, with *a*_s_ the lattice constant of the substrate and *a*_o_ the lattice constant of the overlayer. The misalignment depends on the ratio of the sound velocities *c*_L_ and *c*_T_ of longitudinal acoustic (LA) and transverse acoustic (TA) phonon modes of the adsorbed layer through the parameter.

(3) $$\delta ={\left(c_{L}/c_{T}\right)}^{2}-1$$

Importantly, misalignment occurs only if *ρ* *δ* > 1. For Au(111)/MoS_2_ system, *ρ*  =  2.884/3.14 ≈ 0.918 and *c*_L_/*c*_T_ ≈ 1.565 (Figure S13 in Section S6.3, Supporting Information), and corresponding theoretical misfit angle is *θ*_NM_ ≈ 1.84°. Theoretically, the frictional stress is expected to increase monotonically for twist angles less than 1.8°, and decrease monotonically for twist angles greater than 1.8°.^[^ ^69^ ^]^ However, our calculations show that the frictional stress exhibits an oscillating trend at small twist angles (Figure 4b), which originates from the oscillations of the sliding energy barrier (Figure 4c), with the shear modulus showing similar behavior (Figure 4e). Two factors lead to the deviation from the NM theoretical results. First, MoS_2_ exhibits out‐of‐plane corrugations rather than being completely flat; Second, the Au substrate is not rigid, releasing its contact stress by planar contractions/expansions that accompany the interface moiré.^[^ ^69^, ^70^ ^]^ To elucidate the non‐monotonic twist angle dependence of frictional stress, we computed the variations in binding energy (Figure 3f) and the sliding energy barrier (Figure 4c) as functions of the twist angle. The data show that the absolute binding energy initially increases, reaching a maximum at ∼2°, and then decreases as the twist angle increases further. Notably, the sliding energy barrier remains relatively constant (∼65.13 meV atom^−1^) for twist angles between 0° and 2° and decreases monotonically beyond 2°. This trend closely mirrors the observed non‐monotonic behavior of frictional stress, suggesting that its non‐linearity originates from the inherently non‐linear nature of the sliding energy barrier.

To further explore its underlying mechanism, we conducted a detailed investigation of energy dissipation analysis and their relationship to frictional behavior. Our analysis reveals a complex interplay between twist angle, energy dissipation, and frictional stress. Energy dissipation in the *x* and *z* directions decreases monotonically with increasing twist angle (Figure 4d), and the anomalous friction behavior is caused by energy dissipation in the *y* direction of the Au substrate (Figure S14a,d, Supporting Information). Notably, the energy dissipation analysis suggests that frictional behavior is mainly attributed to out‐of‐plane atomic motion (Figure 4c). The out‐of‐plane corrugation, which is strongly influenced by the moiré superstructure, decays rapidly with increasing interface mismatch angle. This leads to significant anisotropy in the energy dissipation channel. Interestingly, at a twist angle of 30°, which represents a special aligned state, we observe a slight increase in frictional stress, consistent with our out‐of‐plane corrugation results. The shear modulus and energy barrier as functions of twist angle exhibit a similar trend (Figure 4c,e). Additionally, both horizontal and vertical energy dissipation increases with applied load (Figures S14b,c,e,f and S15, Supporting Information), leading to increased frictional stress with respect to the normal load. Similar conclusions can be drawn for the energy barrier with respect to the normal load (Figure 4c). Figure S16 (Supporting Information) displays the lateral force traces corresponding to different loads, two periodicities are observed in the force traces, the smaller period corresponding to atomic features, whereas the larger period matches the period of moiré superstructure.

## 3 Conclusion

In conclusion, we have successfully fabricated pristine monolayer islands of MoS_2_ on an Au(111) substrate under UHV conditions and thoroughly characterized their intrinsic properties. The potential difference between the Au(111)/MoS_2_ heterostructure and bare Au was accurately measured by KPFM and was well captured by our DFT calculations. Based on comprehensive DFT calculations on binding energy curves and sliding potential energy surfaces of various configurations, we developed a SAIP that accurately describes the vdW interlayer interactions within the heterogeneous Au/MoS_2_ junction. This force field successfully reproduces the experimental results, including the out‐of‐plane corrugation of MoS_2_ and the optimal twist angle of 0.45° between the contacting surfaces. Furthermore, we uncovered a peculiar non‐monotonic dependence of friction on the interfacial twist angle in the Au/MoS_2_ heterostructure using this force field. The high shear stress between MoS_2_ and the Au(111) substrate at a small twist angle suggests a potential application of MoS_2_ serving as a robust superlubic coating in practical scenarios. Our research provides reliable experimental and computational methods for the precise investigation of the inherent properties of metal/TMDC heterostructures. These approaches hold significant promise for broader applications in exploring other van der Waals layered materials, paving the way for advances in nanoscale tribology and the development of novel 2D heterostructures.

## 4 Experimental Section

### Sample Preparation

To grow pristine monolayer MoS_2_ on a single crystal gold surface, experiments were conducted in a UHV chamber vented by H_2_S gas (1.0 × 10^−6^ mbar) during the deposition process. Mo atoms were deposited using an electron beam evaporator on a freshly prepared Au(111) surface by several Ar^+^ sputtering and annealing cycles. Subsequently, the sample was annealed to 800 K, facilitating the growth of triangular MoS_2_ flakes on the Au(111) substrate.^[^ ^44^, ^52^, ^53^, ^54^ ^]^ After preparation, the sample was transferred to a low temperature STM and a room temperature AFM system via UHV suitcase for further experimental investigation.

### Kelvin Probe Force Microscopy Measurement

Kelvin probe force microscopy is a spatially resolved and powerful technique that utilizes surface potential and local work function. It minimizes the electrostatic forces between tip and sample at each image point by applying a dc‐voltage (*V*_dc_), thereby providing images of the local CPD. Therefore, *V*_dc_ corresponds to CPD and is correlated with the work function difference between the tip and the sample.^[^ ^71^ ^]^ The magnitude of CPD depends on the material and characteristics of the tip. However, when studying different surface regions with the same probe, the tip potential can be considered as a reference electrode for CPD, allowing for a relative comparison of CPD values recorded in two distinct functionalized areas of the sample. In this case, all the measurements were carried out under UHV conditions and conductive AFM probe (PPP‐NCLR, Nano‐sensors) with a fundamental resonance frequency of *f*_1_ = 160 kHz was utilized for the topographic and CPD acquisitions. KPFM was performed in the so‐called FM‐KPFM mode at the 1^st^ flexural mode using an alternating current (AC) excitation voltage of *V*_AC_ =  700 mV and *f*_AC_ =  200 Hz. AC and direct current (DC) biases were applied to the tip so that a smaller CPD corresponded to a smaller tip work function.

### Density Functional Theory Methods

All DFT calculations were carried out using the Fritz Haber Institute Ab Initio Molecular Simulations (FHI‐AIMS) code.^[^ ^72^ ^]^ The tier 2 basis set was employed, with tight convergence settings, encompassing all grid divisions and a more refined outer grid.^[^ ^73^, ^74^ ^]^ The nonlocal many‐body (MBD‒NL)^[^ ^75^, ^76^ ^]^ method augmented Perdew–Burke–Ernzerhof (PBE)^[^ ^77^ ^]^ functional was used to calculate the BE curves and sliding potential energy surface (PES). In addition, Heyd‐Scuseria‐Ernzerhof hybrid density functional (HSE06)^[^ ^78^, ^79^ ^]^ was used to calculate the BE curves near the equilibrium distance for comparison (Section S5.2, Supporting Information provides a comparative analysis of the results obtained with PBE‐MBD‐NL and HSE06‐MBD‐NL calculations). The values of 10^−3^, 10^−5^, and 10^−6^ eV were adopted as the convergence criteria of the self‐consistency cycle based on the sum of eigenvalues, the charge density, and the total energy, respectively. A reciprocal‐space mesh of *k*‐points is set as 17 × 17 × 1 and 33 × 33 × 1 for calculating the BE curves and sliding PES, respectively. To avoid interactions among periodic images, a vacuum layer, $z_{0}=100$ Å, was implemented along the out‐of‐plane direction (The convergence analyses for *k*‐points and vacuum size were detailed in Section S5.3, Supporting Information).

### Molecular Dynamics Simulations

All MD simulations were performed in the open source code Large‐scale Atomic/Molecular Massively Parallel Simulator (LAMMPS) package.^[^ ^80^ ^]^ Periodic boundary conditions were applied along the in‐plane lateral direction, while open boundary conditions were imposed along the out‐of‐plane direction. To better mimic the realistic tribological scenario in MD simulations, a ∼3 nm thick gold substrate was utilized to ensure a bulk‐like structure similar to experimental configurations. In the constructed supercell, the strain rates for both the Au(111) substrate and MoS_2_ were controlled below 0.5% to mitigate the impact of strain on MD results. Prior to calculating the moiré corrugation of MoS_2_, the Au(111)/MoS_2_ heterostructure underwent fully relaxation. Initially, the constructed Au(111)/MoS_2_ heterostructure was continuously heated to 800 K using the Nosé‐Hoover thermostat, followed by cooling to 300 K over 500000 steps. Subsequently, pressure control was carried out using Nosé‐Hoover barostat, and thermal equilibrium was maintained for 300000 steps under conditions of 300 K and zero pressure.^[^ ^81^ ^]^ Following the annealing process, the structure was optimized using the FIRE algorithm.^[^ ^82^ ^]^ During this process, the size of the simulation box remained constant, with a convergence criterion set at 10^−6^ eV/Å. Upon reaching a local minimum, further relaxation was performed using the conjugate gradient (CG) method, and the constraints on the simulation box were lifted. This FIRE+CG process was repeated 10 times to ensure thorough relaxation of the system. Lastly, the FIRE algorithm was employed once again for the ultimate optimization. All MD simulations were conducted with a time step of 1 fs. The Au‐Au interaction was described by an embedded atom method (EAM) potential,^[^ ^83^, ^84^, ^85^ ^]^ and the intralayer interactions of the S‐S, S‐Mo, and Mo‐Mo atomic pairs are described by modified Stillinger‐Weber three‐body potential (SW/MOD).^[^ ^86^, ^87^ ^]^ The SAIP developed in this study characterizes the interlayer interactions of Au‐S and Au‐Mo atomic pairs.

## Conflict of Interest

The authors declare no conflict of interest.

## Author Contributions

Y.Y. and Y.S. contributed equally to this work. W.O., Y.S., and Z.L. proposed and supervised the project. Y.S., A.H., T.G., and E.M. conceived and designed the experiments. Y.S., S.S., and S.H. performed the experiments and characterizations. W.O. designed the force field and MD simulation setup. Y.Y. carried out the DFT and MD calculations and parameterized the force field. All the authors analyzed the data. Y.Y., Y.S., B.W., and W.O. wrote the paper with input from all the authors.

## Supporting information



Supporting Information

## Data Availability

The data that support the findings of this study are available from the corresponding author upon reasonable request.
