# Supplementary material for: Unraveling the Interfacial Properties of Twisted Single‐Crystal Au(111)/MoS2 Heterostructures: A Pathway to Robust Superlubricity
Source: Adv Sci (Weinh). 2025 Apr 4;12(21):2415884. doi: 10.1002/advs.202415884 (PMC12140326; doi:10.1002/advs.202415884)
Supplement: Supplementary file 1 — Supporting Information [file ADVS-12-2415884-s001.pdf]

ADVANCED SCIENCE
Open Access

Supporting Information

for *Adv. Sci.*, DOI 10.1002/advs.202415884

Unraveling the Interfacial Properties of Twisted Single-Crystal Au(111)/MoS_2 Heterostructures: A Pathway to Robust Superlubricity

Yuanpeng Yao, Yiming Song, Bozhao Wu, Sebastian Scherb, Shuyu Huang, Antoine Hinaut, Thilo Glatzel, Ernst Meyer, Ze Liu* and Wengen Ouyang**

Supporting Information

Unraveling the Interfacial Properties of Twisted Single-Crystal Au(111)/MoS_2 Heterostructures: A Pathway to Robust Superlubricity

Yuanpeng Yao,[1#] Yiming Song,[2#*] Bozhao Wu,[3,4] Sebastian Scherb[2], Shuyu Huang[2], Antoine Hinaut[2], Thilo Glatzel,[2] Ernst Meyer,[2] Ze Liu,[1,5*] and Wengen Ouyang[1,5*]

[1]*Department of Engineering Mechanics, School of Civil Engineering, Wuhan University, Wuhan, Hubei 430072, China*

[2]*Department of Physics, University of Basel, Klingelbergstrasse 82, 4056 Basel, Switzerland*

[3]*College of Science, Wuhan University of Science and Technology, Wuhan 430081, China*

[4]*Hubei Province Key Laboratory of Systems Science in Metallurgical Process, College of Science, Wuhan University of Science and Technology, Wuhan 430081, China*

[5]*State Key Laboratory of Water Resources Engineering and Management, Wuhan University, Wuhan, Hubei, 430072, P.R. China*

[#]*Yuanpeng Yao and Yiming Song contributed equally to this work*

[*]Corresponding author. Email: yiming.song@unibas.ch

[*]Corresponding author. Email: ze.liu@whu.edu.cn

[*]Corresponding author. Email: w.g.ouyang@whu.edu.cn

This supporting information document includes the following sections:

1. Observations of Defects on the Surface
2. Potential Description and Validation
3. The Influence of Temperature on the Optimal Angle
4. Additional Out-of-plane Corrugation Results
5. Density Functional Theory Calculations
6. Frictional Behavior

1. Observations of Defects on the Surface

The perfect and detailed atomic lattice and superstructure of MoS_2 are shown in Figure S1a,b, providing evidence of the meticulous quality of the sample preparation. For comparison, images of the MoS_2 region containing defects are presented here, as well (Figure S1c). Chalcogen vacancies are assumed to be the dominant defect here, which are frequently suggested as the primary defect type in the literature. These vacancies exhibit the lowest formation energy in the absence of other reactants.

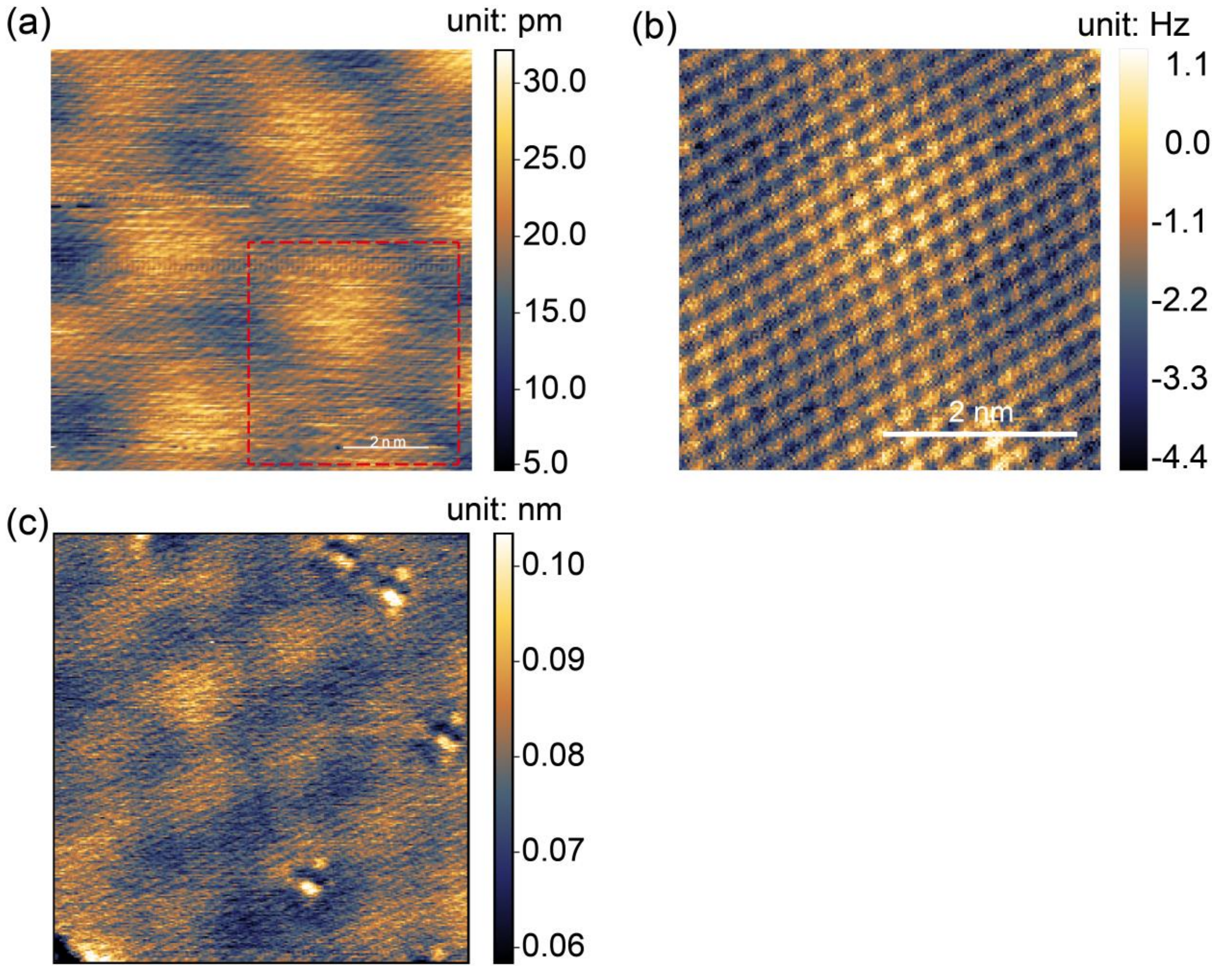

Figure S1. (a) The nc-AFM image showcases the topography of moiré patterns. (b) Zoom-in on the area marked by the red dashed square in panel (a) showing the high-resolution atomic structure of MoS_2 island, as captured by the torsional frequency shift Δf_{TR} recorded in bimodal AFM. (c) Topography of MoS_2 with defects.

2. Potential Description and Validation

2.1 Potential description

This paper adopts a semi-anisotropic interfacial potential (SAIP) model based on the concept of anisotropic interlayer potential in two-dimensional (2D) materials, which consists of a dispersion term and a repulsion term.[1-4] In this model, the dispersion term employs a dispersion correction similar to that developed by Tkatchenko and Scheffler to address long-range van der Waals (vdW) interactions at the interface.[5] The dispersion term is characterized by the utilization of the C_6/r^6 Lennard-Jones (LJ) potential to address long-range vdW interactions, while at short distances, damping is applied using a Fermi-Dirac-type function, resembling the approach introduced in dispersion-corrected density functional theory (DFT) calculations, to prevent the redundant computation of correlation effects[5]

$$E_{\mathrm{dis}}(r_{ij}) = \mathrm{Tap}(r_{ij})\left\{-\frac{1}{1+\mathrm{e}^{-d\left[\left(r_{ij}/\left(s_{\mathrm{R}}\cdot r_{ij}^{\mathrm{eff}}\right)\right)-1\right]}}\cdot\frac{C_{6,ij}}{r_{ij}^{6}}\right\} \tag{S1}$$

where r_{ij} is the distance between i (S or Mo) and j (Au) atom, d and s_R are unit−less parameters determining the steepness and onset of the short-range Fermi-type dampening function. r_{ij}^{eff} and $C_{6,ij}$ are the sum of effective atomic radii and the pair-wise dispersion coefficients, respectively. The $Tap(r_{ij})$ function is typically utilized in calculations for large-scale systems, with the aim of reducing the number of interlayer atomic pairs considered by the system to decrease computational complexity. A continuous long-range cutoff term (up to 3rd derivative) for atomic separations greater than R_{cut} is provided[2]

$$\mathrm{Tap}(r_{ij}) = \frac{20}{R_{\mathrm{cut}}^{7}}r_{ij}^{7} - \frac{70}{R_{\mathrm{cut}}^{6}}r_{ij}^{6} + \frac{84}{R_{\mathrm{cut}}^{5}}r_{ij}^{5} - \frac{35}{R_{\mathrm{cut}}^{4}}r_{ij}^{4} + 1 \tag{S2}$$

The scheme of Kolmogorov-Crespi (KC) potential[1] incorporates isotropic terms with a morse-like exponent multiplied by anisotropic corrections into the repulsive term of the potential energy. The orientation of MoS_2 is described by the normal vectors associated with each S or Mo

$$E_{\mathrm{rep}} = \mathrm{Tap}(r_{ij})\mathrm{e}^{\alpha_{ij}\left(1-\frac{r_{ij}}{\beta_{ij}}\right)}\left\{\varepsilon_{ij} + C\left(\mathrm{e}^{-\left(\frac{\rho_{ij}}{\gamma_{ij}}\right)^{2}} + \mathrm{e}^{-\left(\frac{\rho_{ji}}{\gamma_{ij}}\right)^{2}}\right)\right\} \tag{S3}$$

Here, α_{ij} and β_{ij} serve to modulate the slope and range of potential. C and ε_{ij} represent constant scaling factors measured in energy units. γ_{ij} regulates the width of the Gaussian decay factor within the anisotropic correction term, thereby influencing the repulsive term's sensitivity to lateral distances (ρ_{ij} and ρ_{ji}) between atoms i and j (Figure S2). ρ_{ij} and ρ_{ji} are expressed by the formula of

$$\begin{cases} \rho_{ij}^2 = r_{ij}^2 - (\boldsymbol{n}_i \cdot \boldsymbol{r}_{ij})^2 \\ \rho_{ji}^2 = r_{ij}^2 - (\boldsymbol{n}_j \cdot \boldsymbol{r}_{ij})^2 \end{cases} \tag{S4}$$

where $\boldsymbol{n}_i$ and $\boldsymbol{n}_j$ represent the normalized normal vectors of atoms i (S or Mo) and j (Au), respectively. For the S and Mo atoms in MoS_2, the vector $\boldsymbol{n}_i$ is calculated by averaging the normalized cross products of vectors connecting atom *i* with its three nearest neighbors. Given the isotropy of the electronic cloud of the isolated metal atom (*j*), its normal vector is assumed to lie along the interatomic vector ($\boldsymbol{r}_{ij}$): $\boldsymbol{n}_j \parallel \boldsymbol{r}_{ij}$. In this case, $\rho_{ji} = 0$ can be obtained. Then Eq. (S3) is simplified as

$$E_{\mathrm{rep}} = \mathrm{Tap}(r_{ij}) \mathrm{e}^{\alpha_{ij}\left(1-\frac{r_{ij}}{\beta_{ij}}\right)} \left\{ \varepsilon_{ij} + C \left(\mathrm{e}^{-\left(\frac{\rho_{ij}}{\gamma_{ij}}\right)^2} + 1 \right) \right\} \tag{S5}$$

Finally, the potential function can be expressed as

$$E_{\mathrm{ilp}} = E_{\mathrm{dis}} + E_{\mathrm{rep}} \tag{S6}$$

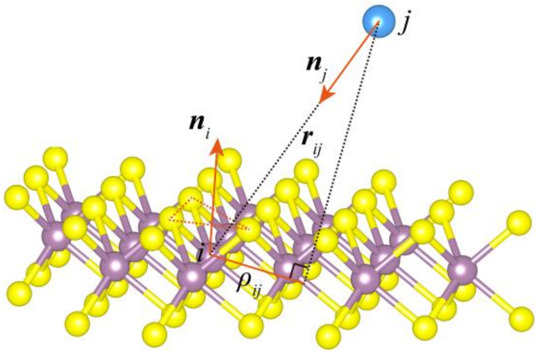

Figure S2. Schematic diagram of normalized normal vectors $\boldsymbol{n}_i$, $\boldsymbol{n}_j$ between i (S or Mo) and j (Au) atom, and their lateral distances ρ_{ij}, ρ_{ji}.

The SAIP parameters are tuned to fit DFT reference datasets by minimizing the following objective function, which measures the disparity between the DFT reference data and the predicted potentials:

$$\Pi(\boldsymbol{\xi}) = \sum_{m=1}^{M_{\mathrm{b}}} w_m^{\mathrm{b}} \left\| E_m^{\mathrm{b}}(\boldsymbol{r}_m, \boldsymbol{\xi}) - E_m^{\mathrm{b,DFT}} \right\|_2 + \sum_{m=1}^{M_{\mathrm{s}}} w_m^{\mathrm{s}} \left\| E_m^{\mathrm{s}}(\boldsymbol{r}_m, \boldsymbol{\xi}) - E_m^{\mathrm{s,DFT}} \right\|_2 \tag{S7}$$

where $\|\cdot\|_2$ is the Euclidean 2-norm. M_{b} and M_{s} are the number of binding energy (BE) curves and sliding potential energy surface (PES), respectively. $E_m^{\mathrm{b/s}}(\boldsymbol{r}_m, \boldsymbol{\xi})$ and $E_m^{\mathrm{b/s,DFT}}$ represents calculated BE/PES using SAIP with parameters $\boldsymbol{\xi}$ and DFT (Experimental Section of main text and Section 5 of Supporting information (SI) gives details of DFT calculations), respectively. w_m^{b} and w_m^{s} are their corresponding weighting coefficients. The spread between the maximum and minimum values of sliding PES is ~66 times the BE. To ensure accuracy, weights are configured as $w_m^{\mathrm{b}} = 66$ and $w_m^{\mathrm{s}} = 1$. The optimization was

conducted utilizing MATLAB with an interior-point algorithm.[6-7] Here, the BE (E_b) is defined as:

$$E_b = \frac{E_t - E_{Au} - E_{MoS_2}}{N_{MoS_2}} \tag{S8}$$

where E_t, E_{Au}, and E_{MoS_2} represents the total energy of the Au/MoS_2, Au(111) substrate and MoS_2, respectively. N_{MoS_2} is the sum of the number of Mo and S atoms.

2.2 Potential validation

Figure S3a,b illustrates the BE curves for Au/MoS_2 calculated using DFT (open symbols) and SAIP (solid lines), with the vertical distance ranging from 2 to 16 Å. The BE, relative error of BE, and equilibrium distance for Au/MoS_2 in different stacking modes are summarized in Table S1. Our computational results indicate a binding energy of ~78 meV/atom between the Au substrate and MoS_2. According to established literature, the boundary between physisorption and chemisorption is typically around 100 meV/atom,[8-9] suggesting that the interaction in our system is best characterized as strong physisorption. This conclusion is further supported by previous studies reporting quasi-covalent characteristics of Au-S bonds,[10-11] which align with our findings. In addition, XPS and Raman data from related studies also indicate strong interactions between the interface atoms of MoS_2 layer and substrate.[12-15] The heterostructure was anchored at the equilibrium distance, and the sliding PES were computed by laterally rigidly displacing MoS_2 that including 256 data points (Figure S3c,d,e). The BE curves and sliding PES demonstrate excellent consistency between SAIP and DFT results, with very small discrepancies. This preliminary evidence supports the accuracy of SAIP (the optimized parameters are listed in Table S2). In addition, we conducted classical molecular dynamics (MD) simulations using SAIP and compared the predicted data with DFT results, as shown in Table S3. The outcomes indicate excellent agreement between the BE and equilibrium distance calculated by SAIP and the DFT results in this study, with errors of 3.1% and 1.7%, respectively. It is important to emphasize that DFT calculations may produce significantly divergent outcomes depending on the choice of functionals, exhibiting fluctuations within the range of ~2.2 to ~266.4 meV/atom (Table S3). The extensive range of fluctuations makes it challenging to determine which functional yields a more reliable BE. Given this, we employed the PBE-MBD-NL method to calculate the potential difference between the bare Au(111) substrate and the Au(111)/MoS_2 surface. The computed result (~315 meV) is in excellent agreement with our KPFM measurements (~320 meV, Figure 1f in main text), providing confirmation of the accuracy in calculating the BE in this work.

Table S1. BE (E_{b}, unit: meV/atom), relative error (ℓ) of BE and equilibrium distance (d_{e}, unit: Å) of Au(111)/MoS_2.

		Type 1	Type 2	Type 3	Type 4
Au(111)/MoS_2	E_b (DFT)	-78.95	-78.73	-78.73	-78.73
	E_b (SAIP)	-78.47	-78.89	-78.89	-78.89
	ℓ (%)	0.61	0.20	0.20	0.20
	d_{e} (DFT)	2.9	2.9	2.9	2.9
	d_{e} (SAIP)	2.9	2.9	2.9	2.9

Table S2. Potential parameters of SAIP for the interfacial Au-S and Au-Mo interactions.

	α	β (Å)	γ (Å)	ε (meV)	C (meV)	d	s_{R}	r^{eff} (Å)	C_6 (eV · Å^6)
Au-S	2.7997	3.2000	1.4444	99.8046	-99.9998	32.2826	2.0000	3.2000	250.0623
Au-Mo	16.8876	4.4427	0.9592	8.6842	31.4345	21.3231	0.9894	4.5386	250.0041

Table S3. Lattice parameters (a), lattice mismatch of Au (MoS_2) (δ), twist angle (θ), equilibrium distance (d_e) and BE (E_b) of Au(111)/MoS_2. Values calculated using the SAIP are compared with first-principles calculations and experimental data.

	Method	a (Å)	δ (%)	θ (°)	d_e (Å)	E_b (meV/atom)
First principles	PW91[16]	5.77	0 (6.09)	30	3.41	-102.33
	vdW-DF[17]	5.53	-4.2 (0)	30	2.9	-36.67
	vdW-DF2[12]			0	2.72	
	LDA[17]	5.53	-4.2 (0)	30	2.6	-30
	LDA[18]	5.40	-6.3 (0)	30	2.92	-133.33
	PBE[17]	5.53	-4.2 (0)	30	3.3	-2.22
	PBE[19]	3.18	10.3 (0)	0	2.51	-83.33
	PBE[19]	11.47	-0.15 (0)	13.9	3.29	
	PBE-D3[20]	5.46	-5.3 (0)	30	2.74	-171.11
	PBE-dDsC[21]	3.08	6.8 (-4.4)	0	2.51	
	PBE-dDsC[21]	11.47	-0.15 (0)	13.9	2.78	-90
	PBE-dDsC[21]	5.77	0 (4.1)	30	2.65	-90
	PBE-MBD-NL (This paper)	5.62	-2.6 (2.93)	30	2.9	-78.95
Experiments	HAADF-STEM[22]				3.5	
MD simulations	LJ[17, 23]	34.12	-1.4 (0)	0	2.75	-58.83
	SAIP (This paper)	34.12	-1.4 (0)	0	2.85	-81.38

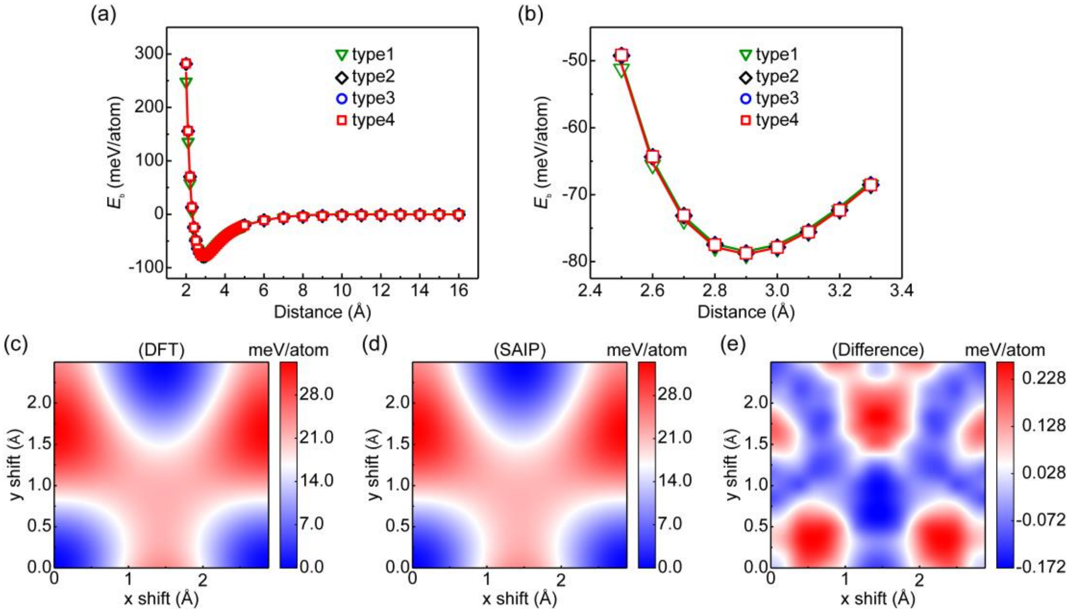

Figure S3. (a) BE curves and (b) zoom-in around the minimum energy of Au(111)/MoS_2 with various stacking modes. Symbols and lines represent DFT and SAIP results, respectively. (c)-(e) Sliding PES of Au(111)/MoS_2 calculated at their equilibrium distance, from left to right are the results of DFT, SAIP and their difference, respectively.

2.3 Calculated moiré superlattice, binding energy and out-of-plane corrugation of Au(111)/MoS_2 heterostructures

In this section, we employ SAIP to calculate the periodicity of moiré superlattice, BE and out-of-plane corrugation with respect to the twist angle. The periodicity of moiré superlattice is determined by the following formula:[24]

$$L_{\text{moiré}} = \frac{a_{\text{Au}}(1+\delta)}{\sqrt{2(1+\delta)(1-\cos\theta)+\delta^2}} \tag{S9}$$

where a_{Au} is the lattice constant of Au substrate, δ and θ are lattice mismatch and twist angles between MoS_2 and the Au substrate, respectively. The moiré superlattice exhibits consistent periodicity with theoretical predictions across various twist angles in the MD simulations (Figure S4). Remarkably, at the twist angle of 30°, we identify a second-order moiré superlattice characterized by a period of ~30.5 Å. It is attributed to the specific geometric relationship, and our previous study provides a detailed explanation.[25]

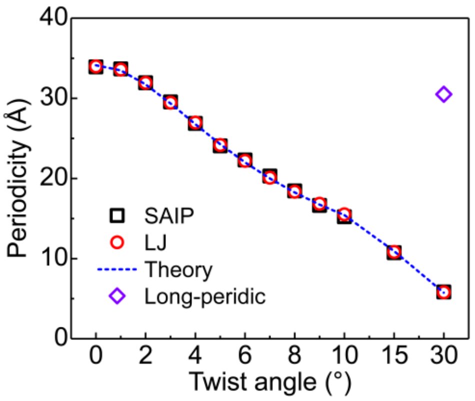

Figure S4. Periodicity of moiré superlattice of Au(111)/MoS_2 with different twist angles. Black and red symbols represent the results of SAIP and LJ, respectively. Purple symbol represents the periodicity of the second-order moiré superlattice calculated by SAIP. Blue dashed line represents the theoretical results obtained through Eq. (S9).

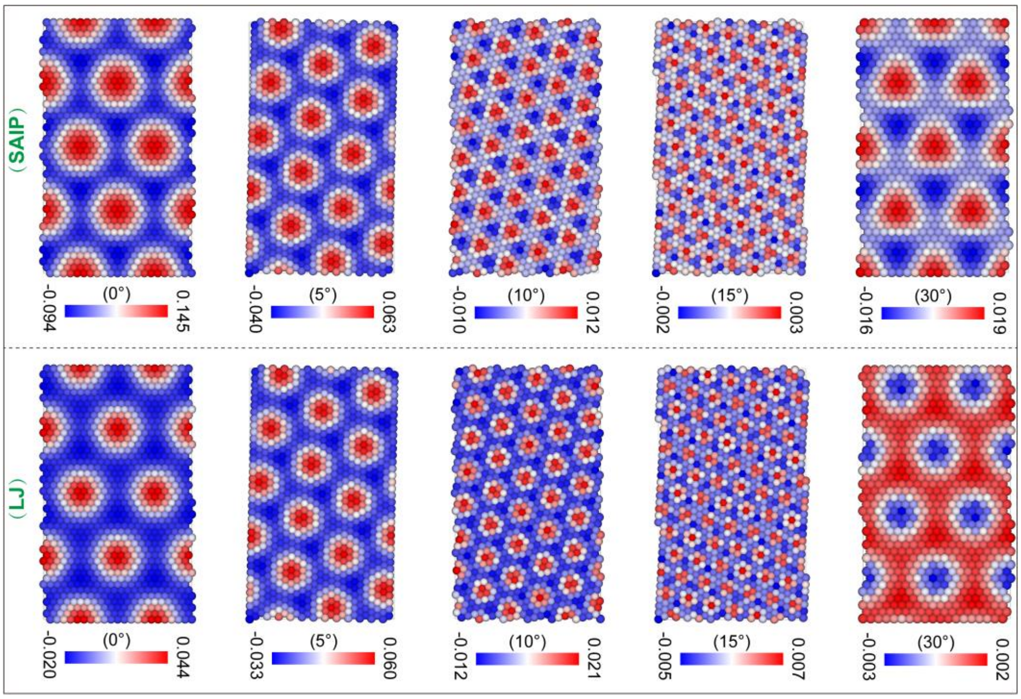

Figure S5. Out-of-plane corrugation of MoS_2 on the substrate of Au(111) with the different twist angle. The first and second rows are the results calculated using SAIP and LJ, respectively.

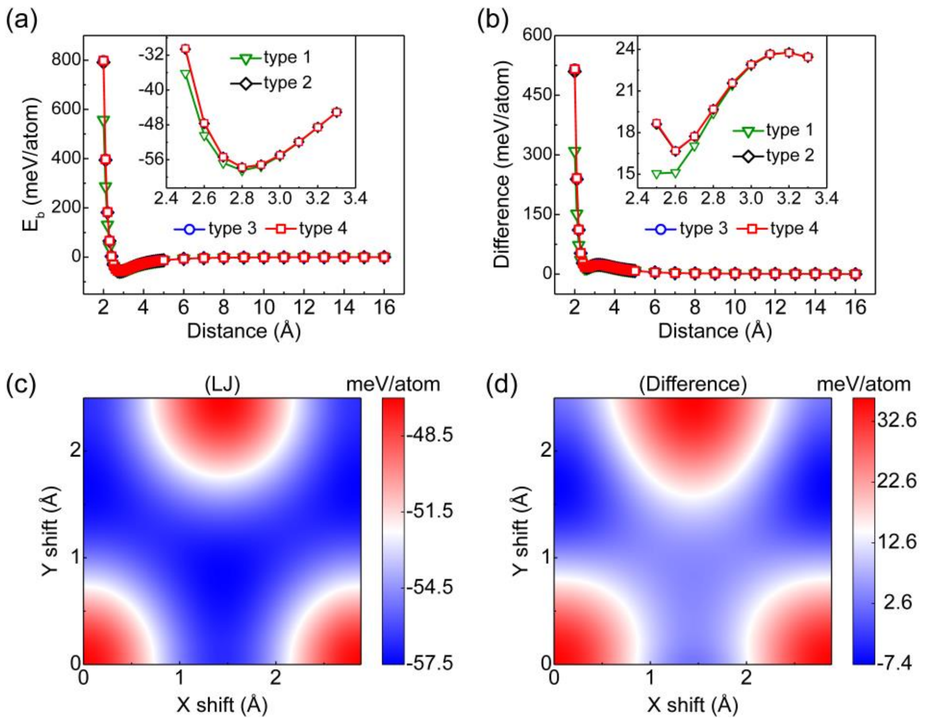

Figure S6. (a, c) BE curves (a) and sliding PES (c) calculated by the LJ potential. Insets provide a magnification around the minimum energy. (b, d) Difference of BE curves (b) and sliding PES (d) between LJ potential and DFT.

2.4 Comparison between semi-anisotropic interfacial potential and Lennard-Jones potential

For comparison with SAIP, we employ the LJ potential with parameters commonly found in the literature ($\epsilon = 0.022$ eV and $\sigma = 2.9$ Å)[17, 23] to describe Au-S and Au-Mo interactions. This allows us to assess the periodicity of the moiré superlattice (Figure S4), out-of-plane corrugation (Figure 3c,d,e, Figure S5 and Figure S9c,d), BE (Figure 3f and Figure S6a,b), sliding PES (Figure S6c,d), optimal angle (Figure 2b,c), interlayer and intralayer energy (Figure S7c,d), and total energy (Figure 2d,e). The LJ potential shows effectiveness in predicting the periodicity of the moiré superlattice. However, the prediction of other properties lacks accuracy, leading to significant differences. This intrinsic limitation hinders its ability to faithfully reproduce experimental results when simulating physical properties like friction, peeling, or thermal conductivity.

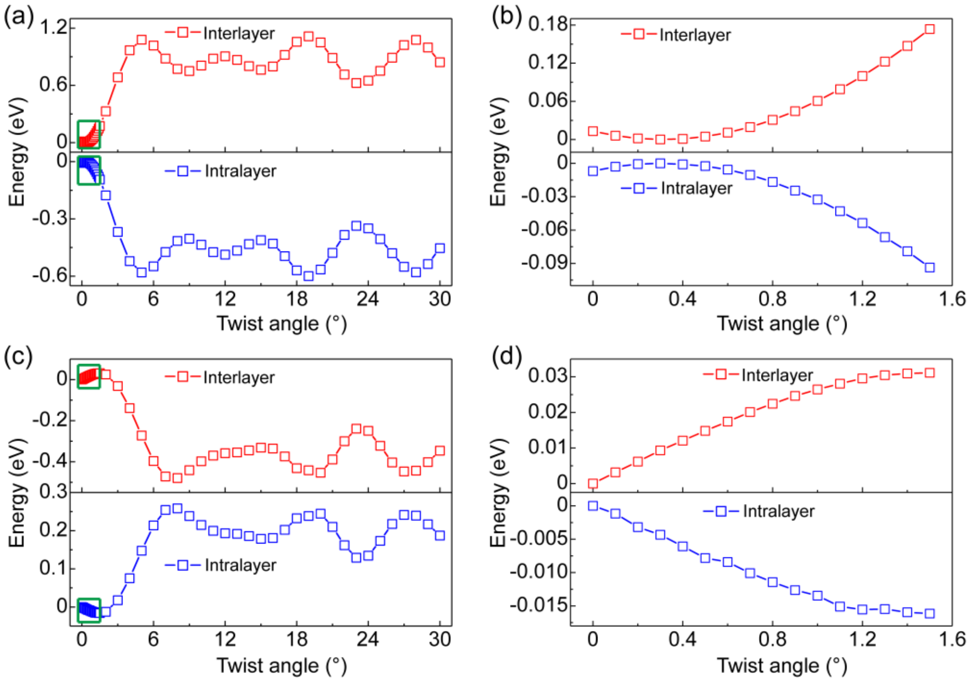

Figure S7. The interlayer energy (red line) and intralayer energy (blue line) as a function of twist angle calculated by (a) SAIP and (c) LJ. (b), (d) Zoomed-in image around 0° in (a) and (c).

3. The Influence of Temperature on the Optimal Angle

To investigate the effect of temperature on the stability of the optimal twist angle, we performed additional molecular dynamics simulations, increasing the temperature of the MoS_2/Au system from 300 K to 500 K (Figure S8). Our results indicate that the optimal twist angle remains stable at 0.47° within a temperature range of 300 K to ~450 K. However, beyond this critical temperature (~450 K), the MoS_2 layer undergoes noticeable rotation, disrupting the optimal twist angle.

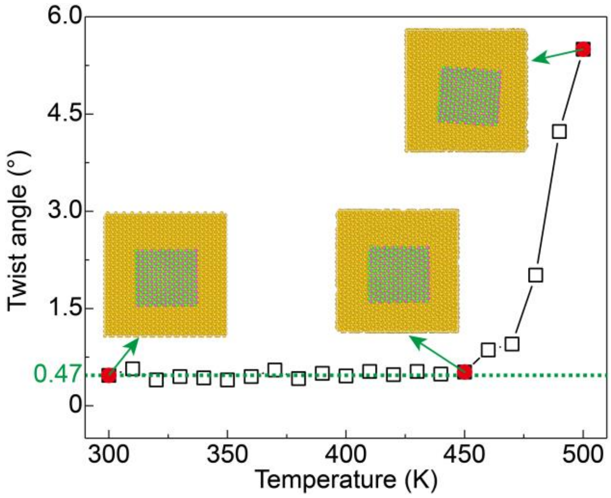

Figure S8. The evolution of the interfacial twist angle as a function of temperature. The green dashed line corresponds to 0.47°, representing the optimal twist angle of the structure. The three red dots represent different states of the system.

4. Additional Out-of-plane Corrugation Results

It's well known that the primary scanning parameters affecting the nc-AFM measurements are amplitude of oscillation A and frequency shift Δf. In Figure 3 of the main text, the cantilever was excited at its first resonance mode, using scanning parameters of Δf_{1st} = -60 Hz and A_{1st} = 5 nm. The resulting height profile clearly reveals the moiré structure, exhibiting a height corrugation within the range of 0.24 ± 0.04 Å. To further investigate the effect of scanning parameters on the measured height profiles, we performed additional nc-AFM measurements, exciting the cantilever at its second resonance mode (Δf_{2nd} = -25 Hz and A_{2nd} = 400 pm, Figure S9). The results demonstrate that the influence of scanning parameters on the nc-AFM height profiles is negligible (Table S4 lists detailed scanning parameters).

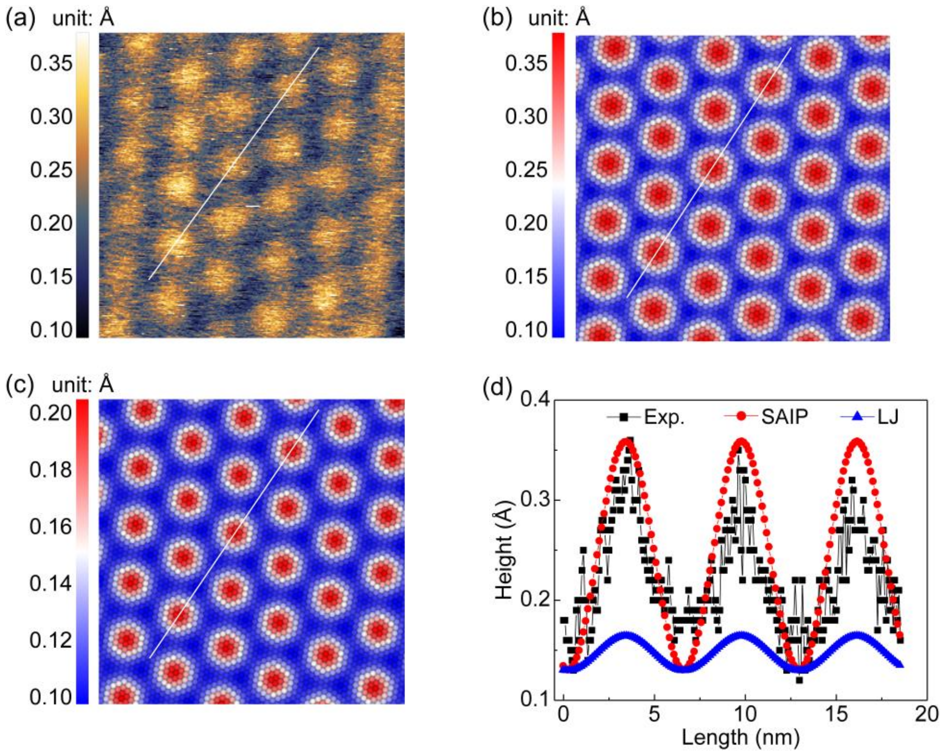

Figure S9. Experimental and simulated moiré superlattice structures of Au/MoS_2 heterostructure. (a) nc-AFM topographic image of the monolayer MoS_2 grown on Au(111) substrate, exhibiting the moiré superlattice. Experimental parameters: Δf_{2nd} = -25 Hz, A_{2nd} = 400 pm. (b, c) Calculated moiré superlattice of Au/MoS_2 with a twist angle of 0.45° using SAIP (b) and LJ potential (c). (d) Height profiles along the white line in (a), (b) and (c), corresponding to black (experiment), red (SAIP), and blue (LJ) lines respectively.

Table S4. Scanning parameters.

Parameters	First resonance mode	Second resonance mode
Resonance Frequency (kHz)	171.6	1049.2
Amplitude (nm)	5	0.4
Δf (Hz)	-60	-25
Scan Speed (nm·s^{-1})	11.3	20

5. Density Functional Theory Calculations

5.1 Density functional theory models

For the Au(111)/MoS_2 heterostructure, the pronounced lattice mismatch requires employing distinct DFT models for accurate computation of BE curves and sliding PES. For the BE curves, $\sqrt{3} \times \sqrt{3}$ MoS_2 (5.46 Å) lattice was matched to 2×2 Au(111) (5.77 Å) lattice to avoid a large mismatch between MoS_2 and Au(111) substrate, and the lattice parameters of Au(111)/MoS_2 are averaged to 5.62 Å, resulting in strains of 2.93% (-2.60%) in MoS_2 (Au) (Figure S10a,b,c,d). For the DFT calculation of the sliding PES, models with higher strains were employed to achieve a corrugated PES. This involved aligning a 1×1 MoS_2 lattice (3.15 Å) with a 1×1 Au(111) lattice (2.88 Å). The averaged lattice parameters were set to 3.02 Å, resulting in strains of -4.13% (4.64%) in MoS_2 (Au) (Figure S10e). To better approximate experimental conditions, a 6-layer Au(111) substrate was used in the DFT model (The impact of varying layers of Au substrate on BE was illustrated in Section 5.2 of SI).

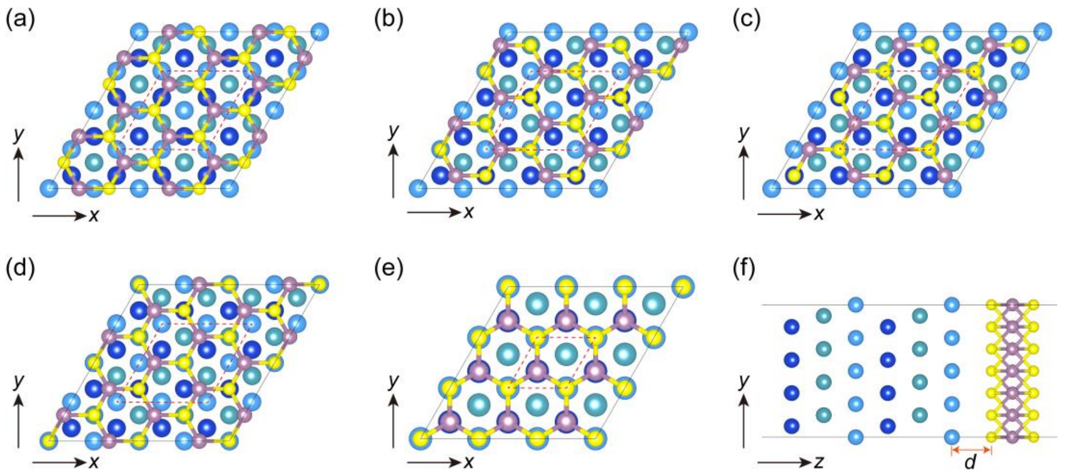

Figure S10. (Color online) Four high-symmetry stacking modes of Au(111)/MoS_2: (a) type 1, (b) type 2, (c) type 3, (d) type 4, and (e) Sliding PES model. (f) Side view. Obtain type 2, type 3 and type 4 by rigidly shifting the MoS_2 in type 1. The unit cell is marked by the red dashed parallelogram. The yellow, brown and other sphere represents S, Mo and Au atoms, respectively.

5.2 Effect of density functional theory method on the binding energy calculations

The DFT calculations in this study were performed using the PBE-MBD-NL method, and their reliability was confirmed through comparison with KPFM. Here, we employed the HSE06-MBD-NL method to calculate the BE curves as a reference (Figure S11), and the results from the two methods are very close.

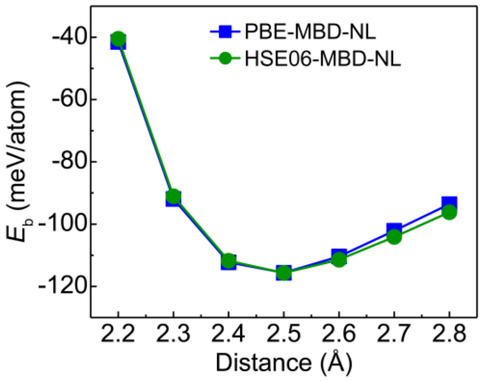

Figure S11. Comparison of BE curves calculated by PBE-MBD-NL (blue line) and HSE06-MBD-NL (green line). The high-strain model was employed for the calculation due to the excessive number of atoms in the low-strain model.

5.3 Convergence tests of the density functional theory calculations

The accuracy of DFT results could be influenced by the *k*-grid density and vacuum size. In this section, we assessed the sensitivity of BE calculations for both high-strain and low-strain models concerning the *k*-grid density and vacuum size (Figure S12a,b,c,d). In each figure, only one parameter varies while keeping the rest consistent with the Experimental Section of density functional theory methods in main text. The red symbols indicate the parameter values used to generate the reference results used in this study. It is evident that for the low-strain model, choosing a 17×17×1 *k*-grid and a vacuum size of 100 Å enhances the convergence accuracy of BE to within ~0.05 meV/atom. For the high-strain model, a 33×33×1 *k*-grid is selected, with the vacuum size remaining at 100 Å. This level of precision meets the requirements of this study. Additionally, considering the potential influence of substrate layers on the results, we examined the trend of BE with varying substrate layers (Figure S12e). The results suggest that the BE tends to stabilize when the substrate is chosen to have 6-layer.

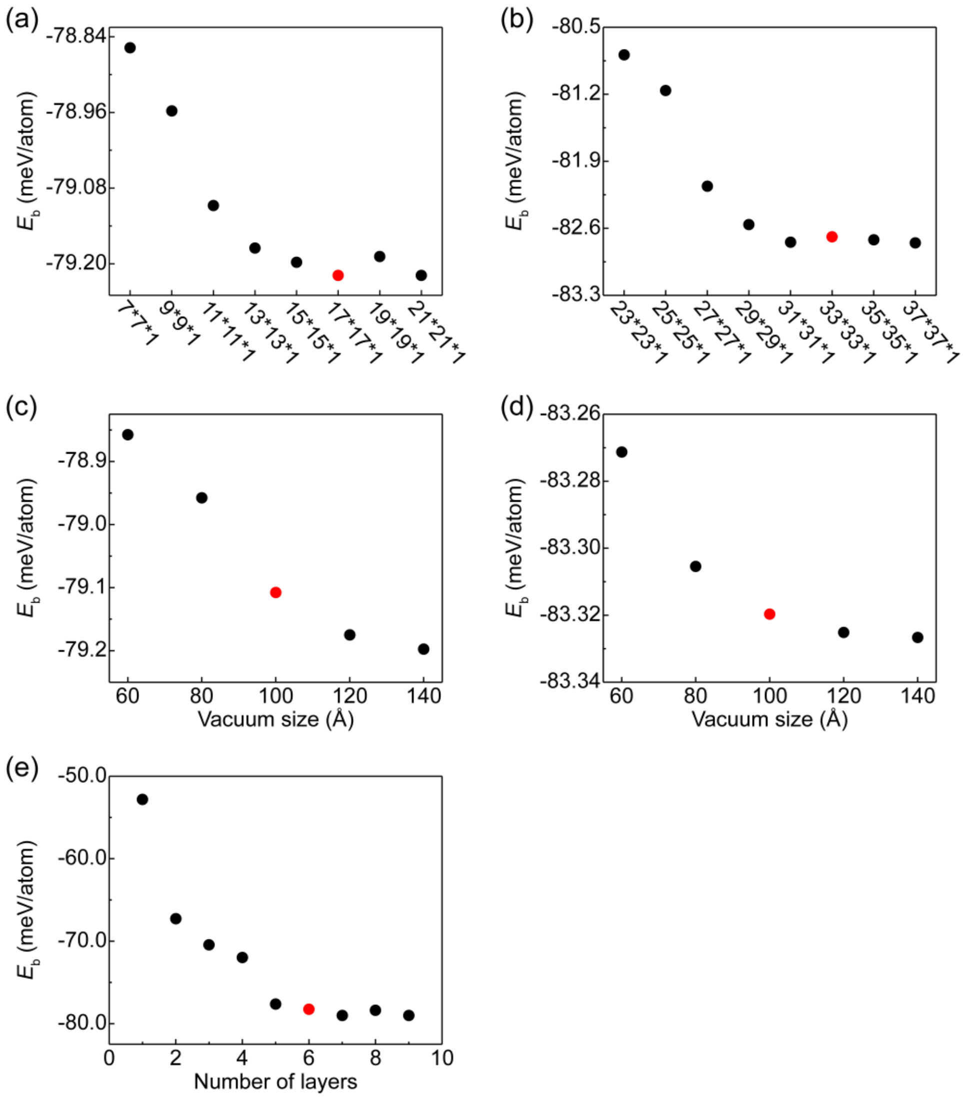

Figure S12. Convergence tests of *k*-grid density, vacuum size and different layers of Au(111) substrate. (a), (c) 2×2 Au(111) matched to $\sqrt{3} \times \sqrt{3}$ MoS_2. (b), (d) 1×1 Au(111) matched to 1×1 MoS_2. The red-colored symbols mark the values used to obtain the results presented in the main text. The distance between Ag(111) and graphene layers is set to 2.9 Å. (e) The impact of different layers of Au substrate on the BE, calculated using model of 2×2 Au(111) matched to $\sqrt{3} \times \sqrt{3}$ MoS_2. Convergence of BE on 6-layer Au substrate.

6. Frictional Behavior

6.1 MD simulations of frictional behavior

A 6-layer Au substrate and a 3-layer MoS_2 constitute the MD model for the evaluation of frictional behavior of the heterogeneous interface, with MoS_2 arranged in an anti-parallel stacking modes (AA').[26] Prior to sliding MoS_2, the system was relaxed for 500,000 steps using the microcanonical ensemble (NVE) combined with the Langevin thermostat. The temperature was controlled at 0 K, and the time step was set to 1 fs. After sufficient relaxation of the atoms, friction simulations were conducted with a modified time step of 0.5 fs to ensure a more detailed capture of lateral force changes. The MoS_2 rigid layer moves along the Au lattice direction through a spring with a constant velocity of $\boldsymbol{v} = 5\ \mathrm{m/s}$. To evacuate the heat generated at the shear interface, velocity damping with a damping coefficient of $\eta_{x0}^{\mathrm{Au}} = \eta_{y0}^{\mathrm{Au}} = \eta_{z0}^{\mathrm{Au}} = \eta_{x0}^{\mathrm{MoS_2}} = \eta_{y0}^{\mathrm{MoS_2}} = \eta_{z0}^{\mathrm{MoS_2}} = 1.0\ \mathrm{ps^{-1}}$ is applied to remote flexible regions of Au(111) and MoS_2 far away from the sliding interface (Figure 4a). The normal load is applied by adding a vertical constant force on each of the atom in the rigid part of the MoS_2 layer which helps spreading the load evenly across the interface, with magnitude of 0, 0.01, and 0.05 nN/atom respectively, corresponding to an overall normal load of 0, 358, and 1790 MPa. All atoms are free to move in any direction, apart from those of the bottommost Au layer, which are held fixed at their equilibrium positions, and those of the moving stage that are rigidly shifted in the *x* direction at a constant velocity. The sliding distance was set to be no less than 25 nm to ensure that the system reached a steady state, and the final 5 nm was used for analysis in this study.

6.2 Evaluation of frictional dissipation

We apply a damping force of $\mathbf{f}_{\mathrm{damp,MoS_2}}^{i}(t)$ and $\mathbf{f}_{\mathrm{damp,Au}}^{i}(t)$ to each atom, i, within the damped regions in the MoS_2 and Au substrate, respectively, using the following equations:

$$\begin{cases} \mathbf{f}_{\mathrm{damp,MoS_2}}^{i}(t) = -m_{\mathrm{MoS_2}}\eta_{x0}^{\mathrm{MoS_2}}\left(v_x^i(t) - v_{0,x}\right)\mathbf{x} - m_{\mathrm{MoS_2}}\eta_{y0}^{\mathrm{MoS_2}}\left(v_y^i(t) - v_{0,y}\right)\mathbf{y} - m_{\mathrm{MoS_2}}\eta_{z0}^{\mathrm{MoS_2}} v_z^i(t)\mathbf{z} \\ \mathbf{f}_{\mathrm{damp,Au}}^{i}(t) = -m_{\mathrm{Au}}\sum_{\alpha=x,y,z}\eta_{\alpha0}^{\mathrm{Au}} v_\alpha^i(t)\,\boldsymbol{\alpha} \end{cases} \quad (\mathrm{S10})$$

Here, $m_{\mathrm{MoS_2}}$ and m_{Au} are the atomic masses of S (or Mo) and Au, respectively, $v_\alpha^i(t)$ is the α Cartesian velocity component of the damped i^{th} atom at time t, $v_{0,x}$ and $v_{0,y}$ are the *x* and *y* components of sliding velocity $\boldsymbol{v}$, respectively, and $\boldsymbol{\alpha} = \mathbf{x}, \mathbf{y}, \mathbf{z}$ are the unit vectors along the Cartesian x, y and z directions, respectively. The equations of motion for the S or Mo atoms are given by

$$m_{\mathrm{MoS_2}}\ddot{\boldsymbol{r}}_i = -\boldsymbol{\nabla}_{\boldsymbol{r}_i}(V_{\mathrm{inter}} + V_{\mathrm{intra}}) + k_{\parallel}\left(\boldsymbol{r}_{\parallel,i}^{\mathrm{stage}} - \boldsymbol{r}_{\parallel,i}\right) - F_{\mathrm{N}}z + \mathbf{f}_{\mathrm{damp,MoS_2}}^{i} \quad (\mathrm{S11})$$

where the first two terms on the right represent the forces generated by interlayer and in-plane interactions, the third term corresponds to the lateral elastic driving force during the sliding stage, the fourth term is the applied normal load, and the last term is the viscous force accounting for energy dissipation needed to reach a steady state.[27] The equations of motion for the Au atoms are given by

$$m_{\mathrm{Au}}\ddot{\boldsymbol{r}}_i = -\boldsymbol{\nabla}_{\boldsymbol{r}_i}(V_{\mathrm{inter}} + V_{\mathrm{intra}}) + \mathbf{f}^{i}_{\mathrm{damp,Au}} \tag{S12}$$

All atoms are free to move in any direction, except for the atoms in the bottommost Au substrate (fixed at equilibrium positions) and the atoms undergoing rigid motion (moving rigidly at a constant velocity in the *x*-direction). For Eq. (S11), V_{inter} and V_{intra} are represented as

$$\begin{cases} V_{\mathrm{inter}} = \mathrm{V}_{\mathrm{inter}}^{\mathrm{SS-SAIP}} + \mathrm{V}_{\mathrm{inter}}^{\mathrm{MoMo-SAIP}} + \mathrm{V}_{\mathrm{inter}}^{\mathrm{SMo-SAIP}} + \mathrm{V}_{\mathrm{inter}}^{\mathrm{AuS-SAIP}} + \mathrm{V}_{\mathrm{inter}}^{\mathrm{AuMo-SAIP}} \\ V_{\mathrm{intra}} = \mathrm{V}_{\mathrm{intra}}^{\mathrm{SW/MOD}} \end{cases} \tag{S13}$$

For Eq. (S12), V_{inter} and V_{intra} are represented as

$$\begin{cases} V_{\mathrm{inter}} = \mathrm{V}_{\mathrm{inter}}^{\mathrm{AuS-SAIP}} + \mathrm{V}_{\mathrm{inter}}^{\mathrm{AuMo-SAIP}} \\ V_{\mathrm{intra}} = \mathrm{V}_{\mathrm{intra}}^{\mathrm{EAM}} \end{cases} \tag{S14}$$

The instantaneous shear-force is given by the sum of the lateral spring forces,

$$F_i(t) = k_{\parallel}\left(\boldsymbol{r}_{\parallel,i}^{\mathrm{stage}} - \boldsymbol{r}_{\parallel,i}\right) \tag{S15}$$

acting between all atoms of the sliding MoS_2 layer and virtual atom within the rigidly moving stage. The kinetic friction force, F_{k}, is evaluated as the time average of the total shear-force acting on the moving stage in the sliding direction,

$$F_{\mathrm{k}} = \langle \sum_{i=1}^{N_{\mathrm{MoS_2}}} F_{\mathrm{x},i}\,(t)\rangle \tag{S16}$$

The time average is taken after the initial transient dynamics decays and the system reaches steady-state motion. Here, $\langle\ \rangle$ denotes a steady-state time average. At steady-state, the power $\langle P_{\mathrm{in}}(\mathrm{t})\rangle$ generated by the external springs equals the power $\langle P_{\mathrm{out}}(\mathrm{t})\rangle$ dissipated by the internal viscous forces. $\langle P_{\mathrm{in}}(\mathrm{t})\rangle$ is given by

$$\langle P_{\mathrm{in}}(\mathrm{t})\rangle = \sum_{i=1}^{N_{\mathrm{MoS_2}}} \langle \boldsymbol{F}_i(t)\cdot \boldsymbol{v}_i(t)\rangle = F_{\mathrm{k}}v \tag{S17}$$

while the second can be written as the sum of contributions coming from each layer,

$$\langle P_{\mathrm{out}}(\mathrm{t})\rangle = \sum_{i=1}^{N_{\mathrm{layer}}} \langle P_{\mathrm{out}}^{\mathrm{i}}(\mathrm{t})\rangle \tag{S18}$$

and $\langle P_{\mathrm{out}}^{\mathrm{i}}(\mathrm{t})\rangle$ is represented as

$$\langle P_{\mathrm{out}}^{\mathrm{i}}(\mathrm{t})\rangle = \sum_{k=1}^{N_{\mathrm{i}}} m_k^i \sum_{\alpha=x,y,z} \left[\eta_\alpha \left\langle \left(v_{\alpha,k}^i(t)\right)^2 \right\rangle\right] = \sum_{k=1}^{N_{\mathrm{i}}} m_k^i \sum_{\alpha=x,y,z} \left[\eta_\alpha \left\langle \left(v_{\alpha,k}^i(t) - v_{\alpha,\mathrm{com}}^i(t)\right)^2 \right\rangle\right] + M_i \sum_{\alpha=x,y,z} \left[\eta_\alpha \left\langle \left(v_{\alpha,\mathrm{com}}^i(t)\right)^2 \right\rangle\right] \quad \text{(S19)}$$

Here, N_i, v_{com}^i and M_i are the total number of atoms in the i-th layer, its center-of-mass velocity and its total mass, respectively. Eq. (S18) and Eq. (S19) leads to the following expression for the kinetic frictional stress:

$$\sigma = \frac{F_{\mathrm{k}}}{A} = \frac{\sum_{i=1}^{N_{\mathrm{layer}}} [\sum_{k=1}^{N_i} m_k \sum_{\alpha=x,y,z} \eta_\alpha \left\langle \left(v_{\alpha,k}^i(t) - v_{\alpha,\mathrm{com}}^i(t)\right)^2 \right\rangle + M_i \sum_{\alpha=x,y,z} \eta_\alpha \left\langle \left(v_{\alpha,\mathrm{com}}^i(t)\right)^2 \right\rangle]}{A \cdot v} \quad \text{(S20)}$$

where A is the contact area.

6.3 Phonon spectra of monolayer MoS_2

To obtain the ratio of the sound velocities c_{L} and c_{T}, the phonon spectra was calculated using the $17 \times 17 \times 1$ supercell of monolayer MoS_2. The interatomic interactions were described by the SW/MOD potential. The MD simulation method is the same as in the Experimental Section of molecular dynamics calculations in main text.

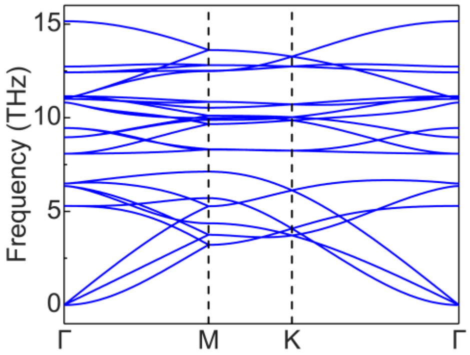

Figure S13. Phonon spectra of monolayer MoS_2 calculated by SW/MOD potential.

6.4 Energy dissipation

From the analysis in the main text, it is evident that the cause of the abnormal frictional stress at small angles lies in the abnormal energy dissipation in the *y* direction of the system (Figure 4d). Here, we further investigate this by analyzing the energy dissipation of the Au substrate and the MoS_2 layer in different directions. The comparison shows that the abnormal energy dissipation in the *y* direction of the Au substrate is responsible for the abnormal frictional stress, while the energy dissipation of the MoS_2 layer, being negligible in proportion, has almost no impact on the overall system (Figure S14a,d). Applying a normal load results in an overall

increase in energy dissipation, but the trend remains unchanged (Figure S14b,c,e,f and Figure S15).

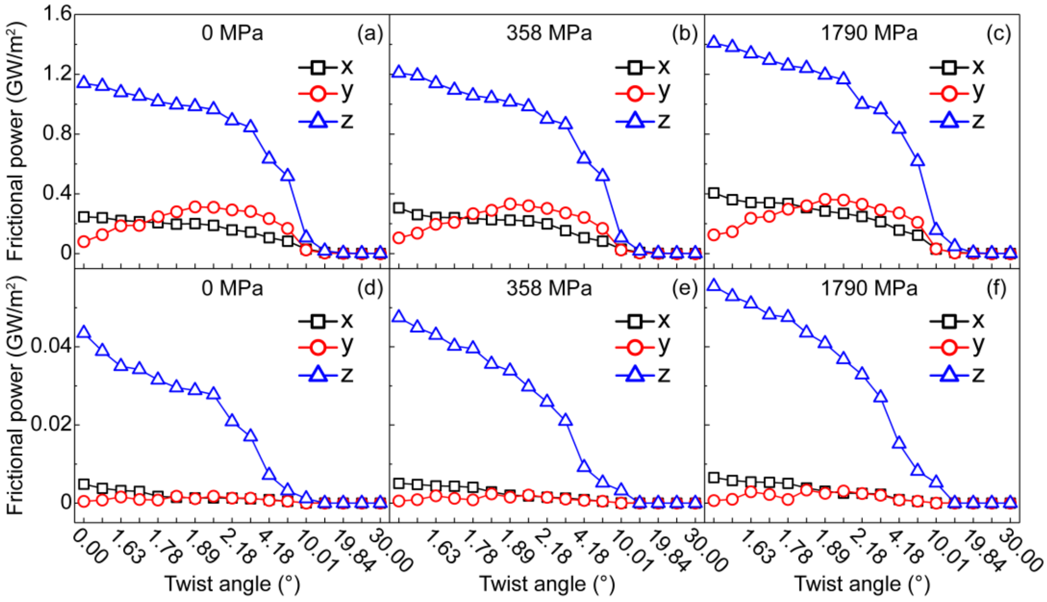

Figure S14. (a)-(c) Energy dissipation along different directions for the (a)-(c) Au(111) substrate and (d)-(f) MoS_2 under various normal loads. The black, red, and blue lines correspond to the *x*, *y*, and *z* directions, respectively.

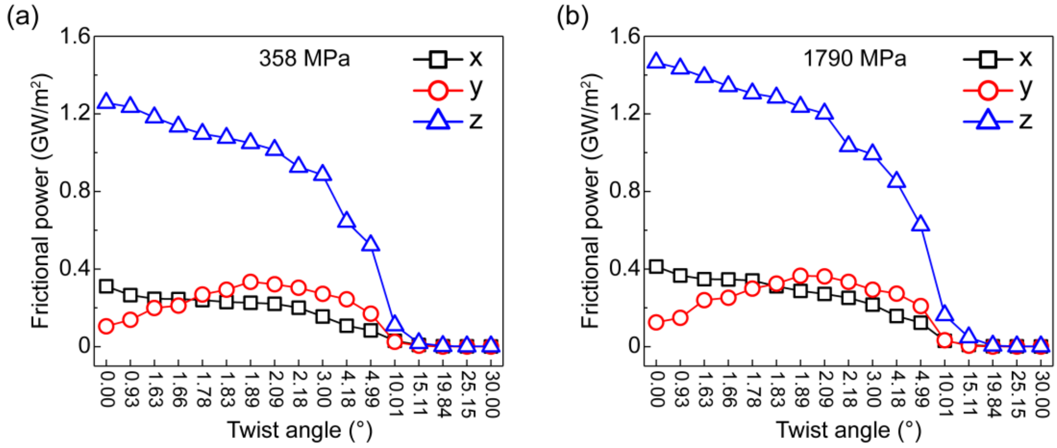

Figure S15. Total dissipation distribution along the *x* (black line), *y* (red line), and *z* (blue line) directions. (a) and (b) correspond to loads of 358 and 1790 MPa, respectively.

6.5 Lateral force traces

Here, the lateral force trace for the Au(111)/MoS_2 system at different twist angles and loads are presented. The plots display two periods: the large period corresponds to the moiré superstructure scale, while the small period corresponds to the atomic scale (Figure S16). As the twist angle increases, the periodicity of the moiré superstructure gradually decreases. The oscillation amplitude of the lateral force trace is positively correlated with the applied load.

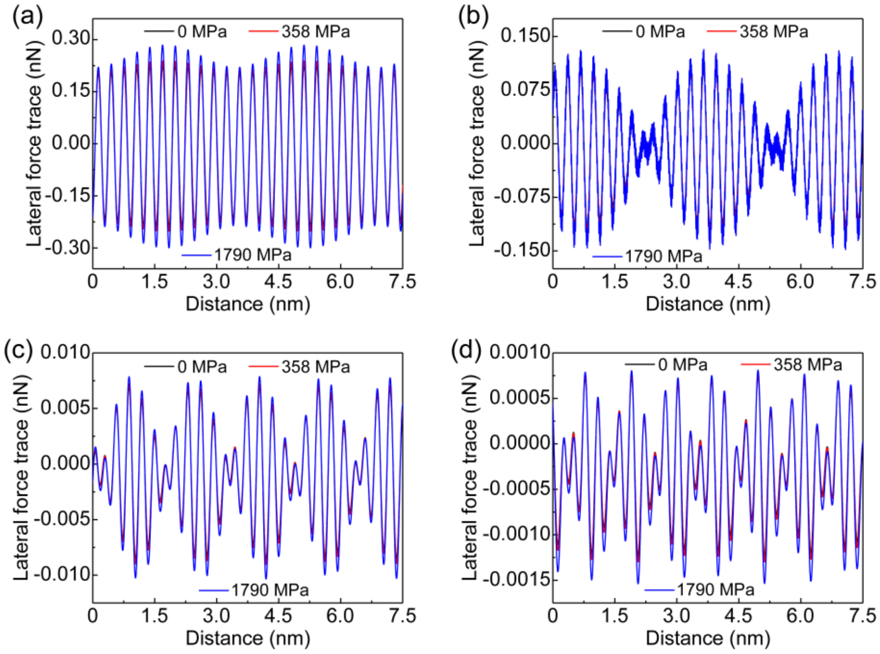

Figure S16. Lateral force traces with different twist angle. (a) 0°, (b) 5°, (c) 10°, and (d) 15°. The loads corresponding to the black, red, and blue lines are 0, 358, and 1790 MPa, respectively.

References

[1] A. N. Kolmogorov, V. H. Crespi, *Phys. Rev. B* **2005**, *71*, 235415.

[2] I. Leven, I. Azuri, L. Kronik, O. Hod, *J. Chem. Phys.* **2014**, *140*, 104106.

[3] I. Leven, T. Maaravi, I. Azuri, L. Kronik, O. Hod, *J. Chem. Theory Comput.* **2016**, *12*, 2896-2905.

[4] T. Maaravi, I. Leven, I. Azuri, L. Kronik, O. Hod, *J. Phys. Chem. C* **2017**, *121*, 22826-22835.

[5] A. Tkatchenko, M. Scheffler, *Phys. Rev. Lett.* **2009**, *102*, 073005.

[6] R. A. Waltz, J. L. Morales, J. Nocedal, D. Orban, *Math. Program.* **2005**, *107*, 391-408.

[7] R. H. Byrd, J. C. Gilbert, J. Nocedal, *Math. Program.* **2000**, *89*, 149-185.

[8] F. Huber, J. Berwanger, S. Polesya, S. Mankovsky, H. Ebert, F. J. Giessibl, *Science* **2019**, *366*, 235-238.

[9] D. Borodin, I. Rahinov, P. R. Shirhatti, M. Huang, A. Kandratsenka, D. J. Auerbach, T. Zhong, H. Guo, D. Schwarzer, T. N. Kitsopoulos, *et al*, *Science* **2020**, *369*, 1461-1465.

[10] S. B. Desai, S. R. Madhvapathy, M. Amani, D. Kiriya, M. Hettick, M. Tosun, Y. Zhou, M. Dubey, J. W. A. III, D. Chrzan, *et al*, *Adv. Mater.* **2016**, *28*, 4053-4058.

[11] Y. Huang, Y.-H. Pan, R. Yang, L.-H. Bao, L. Meng, H.-L. Luo, Y.-Q. Cai, G.-D. Liu, W.-J. Zhao, Z. Zhou, *et al*, *Nat. Commun.* **2020**, *11*, 2453.

[12] C. C. Silva, D. Dombrowski, N. Atodiresei, W. Jolie, F. F. z. Hagen, J. Cai, P. T. P. Ryan, P. K. Thakur, V. Caciuc, S. Blügel, *et al*, *2D Mater.* **2022**, *9*, 025003.

[13] S. G. Sørensen, H. G. Füchtbauer, A. K. Tuxen, A. S. Walton, J. V. Lauritsen, *ACS Nano* **2014**, *8*, 6788-6796.

[14] S. S. Grønborg, S. Ulstrup, M. Bianchi, M. Dendzik, C. E. Sanders, J. V. Lauritsen, P. Hofmann, J. A. Miwa, *Langmuir* **2015**, *31*, 9700-9706.

[15] Y. Bao, J. Shao, H. Xu, J. Yan, P.-T. Jing, J. Xu, D. Zhan, B. Li, K. Liu, L. Liu, *et al*, *ACS Nano* **2024**, *18*, 27411-27419.

[16] H. Zhong, R. Quhe, Y. Wang, Z. Ni, M. Ye, Z. Song, Y. Pan, J. Yang, L. Yang, M. Lei, *et al*, *Sci. Rep.* **2016**, *6*, 21786.

[17] M. Farmanbar, G. Brocks, *Phys. Rev. B* **2016**, *93*, 085304.

[18] C. Gong, L. Colombo, R. M. Wallace, K. Cho, *Nano Lett.* **2014**, *14*, 1714-1720.

[19] A. Bruix, J. A. Miwa, N. Hauptmann, D. Wegner, S. Ulstrup, S. S. Grønborg, C. E. Sanders, M. Dendzik, A. G. Čabo, M. Bianchi, *et al*, *Phys. Rev. B* **2016**, *93*, 165422.

[20] K.-A. Min, J. Park, R. M. Wallace, K. Cho, S. Hong, *2D Mater.* **2016**, *4*, 015019.

[21] S. Sarkar, P. Kratzer, *J. Phys. Chem. C* **2021**, *125*, 26645-26651.

[22] M. Velický, G. E. Donnelly, W. R. Hendren, S. McFarland, D. Scullion, W. J. I. DeBenedetti, G. C. Correa, Y. Han, A. J. Wain, M. A. Hines, *et al*, *ACS Nano* **2018**, *12*, 10463-10472.

[23] F. Trillitzsch, R. Guerra, A. Janas, N. Manini, F. Krok, E. Gnecco, *Phys. Rev. B* **2018**, *98*, 165417.

[24] K. Wang, C. Qu, J. Wang, W. Ouyang, M. Ma, Q. Zheng, *ACS Appl. Mater. Interfaces* **2019**, *11*, 36169-36176.

[25] Y. Yao, B. Wu, Z. Liu, W. Ouyang, *J. Phys. Chem. C* **2024**, *128*, 6836-6851.

[26] W. Ouyang, R. Sofer, X. Gao, J. Hermann, A. Tkatchenko, L. Kronik, M. Urbakh, O. Hod, *J. Chem. Theory Comput.* **2021**, *17*, 7237-7245.

[27] D. Mandelli, W. Ouyang, O. Hod, M. Urbakh, *Phys. Rev. Lett.* **2019**, *122*, 076102.
